# Incidence of Major Depressive Disorder Relapse and Effectiveness of Pharmacologic and Psychological Interventions in Primary Care: A Systematic Review and Meta-Analysis: Incidence de la rechute du trouble dépressif majeur et efficacité des interventions pharmacologiques et psychologiques en soins primaires : revue systématique et méta-analyse

**DOI:** 10.1177/07067437251322401

**Published:** 2025-03-17

**Authors:** Waseem Abu-Ashour, Stephanie Delaney, Alison Farrell, John-Michael Gamble, John Hawboldt, Joanna E. M. Sale

**Affiliations:** 1 School of Pharmacy, 7512 Memorial University, Health Sciences Centre, St. John's, NL, Canada; 2 102793 NL Health Services, Eastern Health, St. John's, NL, Canada; 3 12360 Faculty of Medicine, Memorial University, St. John's, NL, Canada; 4 School of Pharmacy, 8430 University of Waterloo, Kitchener, ON, Canada; 5 518773 Li Ka Shing Knowledge Institute, St. Michael's Hospital, Unity Health Toronto, Toronto, ON, Canada; 6 Institute of Health Policy, Management & Evaluation, University of Toronto, Toronto, ON, Canada; 7 Department of Surgery, Faculty of Medicine, University of Toronto, Toronto, ON, Canada

**Keywords:** depression, relapse, meta-analysis, primary care, therapy, incidence

## Abstract

**Objective:**

This research aims to investigate the relapse rates of major depressive disorder (MDD) within primary care and evaluate the efficacy of relapse prevention therapies. Despite primary care being the common point of contact for MDD patients, there are limited studies around this.

**Methods:**

We included randomized controlled trials and observational studies examining MDD relapse incidence and the effect of pharmacological and non-pharmacological interventions in preventing relapse in primary care. Databases; Medline via Ovid, EMBASE, The Cochrane Library, PsycInfo (ebsco), and Clinical Trials.gov were searched from their inception until September 7, 2022. Joanna Briggs Institute (JBI) appraisal instrument for methodological quality assessment was used. A proportional data analysis estimated the MDD relapse incidence. Therapy effectiveness results were shown as odds ratios with 95% confidence intervals, with heterogeneity explored via subgroup analysis.

**Results:**

Out of the reviewed studies, 35 met the eligibility criteria. Quality appraisal scores varied between 73% and 96%. MDD relapse incidence was divided into subgroups, revealing that both pharmacotherapy and non-pharmacotherapy led to a similar decrease in relapse rates with combination therapies showing further reduction in relapse. Subgroup analyses by study design, follow-up length, date of study and quality of study also yielded noteworthy findings.

**Conclusion:**

Our findings showed that MDD relapse rates in primary care settings can be effectively reduced by pharmacotherapy, non-pharmacotherapy, or combination therapy. Some psychological interventions might also reduce relapse likelihood. More studies are needed on individual and combined treatments over longer periods to understand their long-term impacts on MDD relapse in primary care.

## Plain Language Summary Title

How Often Depression Returns and How Well Treatments Work in Primary Care: A Review of Studies

Plain Language Summary

This study looked at how often people depression experience return of symptoms, when they are treated in primary care settings, such as family doctor clinics, and how well different treatments work to prevent this return of symptoms. Even though primary care is where many people with depression first seek help, there hasn't been much research on how to best prevent this return of symptoms in this setting. To gather information, the study looked at and summarized the studies that were published around this topic following a standard procedure. The study found that both medication and non-medication treatments were beneficial in reducing the chances of return of depression symptoms. Furthermore, using a combination of treatments was even more beneficial. The study also found interesting results when looking at different factors like the type of study and follow-up duration. In conclusion, this study suggests that various treatment options can effectively reduce return of depression symptoms in primary care. This includes medications, psychological therapies, or a combination of both. However, more research is needed to better understand how these treatments work over longer periods and their long-term effects people with depression.

## Résumé

**Objectif:**

Cette recherche porte sur les taux de rechute du trouble dépressif majeur dans le contexte des soins primaires, ainsi que sur l'efficacité des traitements visant à prévenir ces rechutes. Les soins primaires constituent souvent la porte d'entrée pour les personnes souffrant d'un trouble dépressif majeur, mais il existe peu d'études sur ce sujet.

**Méthode:**

Nous avons inclus des essais contrôlés randomisés et des études d'observation examinant l'incidence des rechutes du trouble dépressif majeur et l'efficacité des interventions pharmacologiques et non pharmacologiques dans la prévention des rechutes dans les soins primaires. Pour ce faire, nous avons consulté les bases de données suivantes : Medline via Ovid, EMBASE, The Cochrane Library, PsycInfo (ebsco) et Clinical Trials.gov, à partir de leur création jusqu'au 7 septembre 2022. L'évaluation de la qualité méthodologique a été réalisée grâce à l'instrument développé par le Joanna Briggs Institute (JBI). Une analyse des données proportionnelles a permis d'estimer l'incidence des rechutes du trouble dépressif majeur. Les résultats de l'efficacité du traitement ont été présentés sous forme de rapports de cotes avec intervalles de confiance à 95 %, l'hétérogénéité ayant été étudiée à l'aide d'une analyse de sous-groupes.

**Résultats:**

Au total, 35 des études examinées répondaient aux critères d'admissibilité. Les résultats des évaluations de la qualité se situent entre 73 % et 96 %. L'incidence des rechutes du trouble dépressif majeur a été divisée en sous-groupes, révélant que les interventions pharmacologiques et les interventions non pharmacologiques entraînent une diminution comparable du taux de rechute. Les traitements combinés ont quant à eux montré une réduction encore plus importante des rechutes. Les analyses de sous-groupes selon la conception de l'étude, la durée du suivi, la date de l'étude et la qualité de l'étude ont aussi donné des résultats intéressants.

**Conclusions:**

Nos résultats montrent que les taux de rechute du trouble dépressif majeur en contexte de soins primaires peuvent être efficacement réduits par la pharmacothérapie, les traitements non pharmacologiques ou les traitements combinés. Certaines interventions psychologiques pourraient également réduire la probabilité de rechute. Il est nécessaire de réaliser des études sur des périodes plus longues sur ces modalités thérapeutiques afin de comprendre leurs effets à long terme sur les rechutes du trouble dépressif majeur en contexte des soins primaires.

## Background

Major depressive disorder (MDD) is the leading cause of disability worldwide with the lifetime prevalence ranging from 2% to 21% of adults experiencing 1 or more episodes of depression.^1^ According to the 2019 Global Burden of Diseases, Injuries, and Risk Factors Study, both depressive and anxiety disorders ranked among the top 25 leading causes of global burden, with MDD being the most disabling of the 2 disorders.^2,3^ Previous research has demonstrated that depression negatively impacts work productivity^3^ and leads to increased healthcare resource utilization.^4,5^

Guideline-recommended treatments for MDD include pharmacologic and psychotherapeutic interventions using a collaborative model of care. Although acute phase treatment (defined as 8–12 weeks of treatment)^6^ has been well established and effectively alleviates depression symptoms, the risk of relapse remains a significant issue.^7^ MDD is highly recurrent,^8^ making relapse prevention 1 of the most critical challenges in managing the disorder.^9^ Studies suggest that 30–85% of patients experience MDD relapse despite antidepressant therapy,^10,11^ with risk factors including residual symptoms (odds ratio (OR) 2.77, 95% confidence interval (CI) [2.08–3.68]), a history of previous relapse (OR 1.69, 95%CI [1.28–2.24]), a history of or current comorbid anxiety disorder (OR 1.74, 95%CI [1.37–2.22]), a history of child maltreatment or abuse (OR 1.5, 95%CI [1.27–1.77]).^12^ Thus, the relapse of depressive symptoms after successful acute MDD treatment is not only common but also a considerable clinical concern.

Frank et al.^13^ proposed a definition of MDD relapse, conceptualizing it as a return of symptoms satisfying the full syndrome criteria for an episode occurring during remission. A systematic review examining the evidence surrounding these concepts and definitions found that specific duration thresholds separating remission from recovery were not meaningful in clinical practice.^14^ In light of this finding and guided by clinical guidelines,^6^ we consider both relapse and recurrence as relapse in this study.

The risk of relapse can be minimized by continuing antidepressant use after acute treatment. Numerous treatment guidelines advise patients with an MDD episode to continue antidepressant therapy for 4–12 months after successful acute phase treatment to prevent relapse and to maintain full therapeutic doses for up to 2 years or more for those at an increased risk of MDD relapse. Nonpharmacological interventions, such as cognitive behavioral therapy (CBT), have also been explored for relapse prevention and found to be beneficial.^15^

Primary care has been defined as the provision of integrated, accessible healthcare services by clinicians who are accountable for addressing a large majority of personal healthcare needs, developing a sustained partnership with patients, and practicing in the context of family and community.^16^ The majority of people with mental health problems are managed in primary care.^17^ Despite primary care being the most frequent point of contact for MDD patients seeking healthcare services, studies examining MDD, therapy, and other critical factors within the primary care context are relatively scarce. The majority of work exploring the scope of the problem of depressive relapse has been done in secondary care settings and is likely to be of limited applicability to primary care,^12^ which is where the vast majority of patients with depression are managed. For instance, there are few investigations into MDD relapse therapy in primary care settings. A systematic review by Prieto-Vila et al.^18^ explored relapse risk factors in MDD patients, but the review only encompassed randomized controlled trials (RCTs) involving psychotherapy. Furthermore, only a few studies have aimed to meta-analyze both pharmacotherapy and non-pharmacotherapy in MDD relapse in primary care. Sim et al.^11^ separately examined pharmacotherapy and non-pharmacotherapy interventions for MDD relapse, including various interventions and pooling heterogeneous designs. However, their studies were drawn from mixed healthcare settings and were not specific to primary care. Another meta-analysis by Gili^19^ identified a small number of RCTs examining relapse prevention, specifically focusing on primary care samples. Since both studies were published in 2015, more primary care studies, including RCTs, have emerged.

Given the aforementioned limitations of the previous literature, we conducted a systematic review and meta-analysis to (1) investigate the incidence rate of relapse episodes for all intervention groups in patients with MDD in primary care settings, and (2) evaluate the effectiveness of pharmacotherapy and/or non-pharmacotherapy interventions to prevent relapse.

## Methods

### Study Design

A systematic review was conducted according to PRISMA (Preferred Reporting Items for Systematic Reviews and Meta-analyses) and MOOSE (Meta-analysis of Observational Studies in Epidemiology) guidelines during all stages of design, implementation and reporting.^20,21^ The systematic review was registered in the PROSPERO international prospective register of systematic reviews (protocol number CRD42021290452).

### Eligibility Criteria

This review included RCTs and nonrandomized controlled trials (non-RCTs), assessing the incidence rate of relapse episodes in adult patients with MDD and the effectiveness of pharmacotherapy and/or non-pharmacotherapy interventions in primary care setting in preventing relapse. Specific study designs that were eligible included (a) RCTs, (b) non-RCTs, or other quasi-experimental studies, (c) cohort studies (CS), (d) case-control studies (CCS), nested case-control or case-cohort studies. Other study designs were excluded from this review, such as systematic reviews and meta-analyses, case series, case reports, or cross-sectional studies because temporality between exposure and outcome was uncertain.^22^

Inclusion criteria for the study population were as follows: (a) adults aged 18 years and older; (b) adults with any type of depressive disorders confirmed by any validated depression scale or by the ICD-10 or the DSM-IV-TR criteria; (c) interventions that included pharmacotherapy, non-pharmacotherapy, psychotherapy or sequential therapy used in MDD. There was no restriction on the type of comparison among intervention; (d) authors’ definition of a primary care setting. Primary care setting could include general practitioner or family physician, nurse practitioner, pharmacists, or mental health worker.

The primary outcome of interest was the incidence of a relapse, measured by any validated depression scale, or changes in the severity of depressive symptoms, as assessed against diagnostic criteria (such as the ICD-10 or the DSM-IV-TR). We excluded studies that included patients with treatment-resistant depression, inpatients and patients who did not receive an intervention or an exposure for depression therapy.

### Data Sources and Searches

The search strategy was carried out in collaboration with a research librarian (AF) experienced in systematic reviews from the date of the databases’ inception to September 7, 2022. We searched the following 5 biomedical databases: Medline via Ovid, EMBASE, The Cochrane Library, PsycInfo (ebsco) and Clinical Trials.gov.

The search was developed in Ovid Medline using MeSH Terms and keywords and was subsequently translated into the other databases, using each database's controlled vocabulary and keywords. There were no restrictions on language in the search; however, only English language studies were included. Grey literature such as information produced on all levels of government, academics, business, and industry in electronic and print formats not controlled by commercial publishing was also searched. Duplicate studies were removed using Endnote ×9 and imported into Covidence for screening. See Supplement File 1 for the specific search strategy used in each database and a list of the grey literature sources.

### Study Selection

Two reviewers (WAA, SD) independently scanned study titles, abstracts, and keywords of every non-duplicate record retrieved from the literature search. Following title/abstract screening, 2 reviewers independently screened the full text of the included articles. Relevant studies were identified using the software Covidence (Covidence systematic review software, Veritas Health Innovation, Melbourne, Australia). Discrepancies were resolved by consensus of the 2 reviewers. If consensus was not achieved, a third reviewer was consulted.

### Data Extraction and Quality Assessment

Data were extracted by 1 reviewer (WAA) onto a predesigned form that included first author, contact details, journal citation, year of publication, study design, funding, sample size, data set used, study location and duration, duration of follow-up, study population characteristics, statistical analysis used, depression diagnosis tool used, relapse definition, the study-specific measure of association, and type of intervention and exposure. Studies with confirmed inclusion criteria were assessed for methodological quality using the standardized critical appraisal instrument from the Joanna Briggs Institute (JBI) Meta-Analysis of Statistics Assessment and Review Instrument.^23^ The instrument chosen for review was dependent on the study design of each study included. Each criterion was allocated a score (Yes = 2, No = 0, Unclear = 1), giving a total possible score of 26 for RCTs, a score of 22 for cohort studies and a score of 20 for case-control studies. These scores were then converted to a percentage^24,25^ for ease of interpretation and comparison. Guided by previous studies using a similar approach in quality assessment,^24,25^ the quality of each review was ranked based on the following criteria: 0–33% of criteria met (low quality), 34–69% of criteria met (medium quality), and 70% or more of criteria met (high quality). The study selection process was conducted independently by 2 reviewers (WAA, SD). Any disagreements were resolved through discussion, or with a third reviewer (JH). Moreover, included studies that had more than 2 treatment arms, were shown in the forest plots as “author, year, arm—portrayed as a number [1,2,3, etc.].”

### Data Synthesis and Analysis

Characteristics of included studies were described and stratified by study design. To estimate the incidence of MDD relapse in this study, we conducted a proportional data analysis. When pooling proportions for meta-analysis, a transformation of the data is required. To transform the data, we used the logit transformation.^26,27^ This method is used to calculate the weighted summary proportion under the random-effects model. Random effects models were used to pool results from included studies for the outcome.^28^ The resultant meta-analysis gives a pooled proportion with a 95% CI both for the fixed and the random-effects model, and lists the proportions (expressed as a percentage), with their 95% CI, found in the individual studies included in the meta-analysis. The results were graphically presented in a forest plot. For the effectiveness of therapies, the results were expressed as an OR with corresponding 95% CI. Heterogeneity was assessed using the Chi-Squared test and measured using the I-squared statistic as recommended by the Cochrane handbook. We followed the guide provided by the Cochrane handbook on the interpretation of heterogeneity.^28^ We considered heterogeneity to be “not important” if the I-squared statistic was between 0% and 40%; “moderate heterogeneity” if the I-squared statistic was between 30% and 60%; “substantial heterogeneity” if the I-squared statistic was between 50% and 90%; and “considerable heterogeneity” if the I-squared statistic was between 75% and 100%. Heterogeneity was explored using subgroup analysis.^28,29^ Meta-analysis was conducted for all treatment arms combined to measure the incidence summary proportion. Meta-analysis was also conducted for subgroups of pharmacotherapy, non-pharmacotherapy and combination therapy. Furthermore, subgroup metanalysis was conducted according to the study design. Finally, we meta-analyzed the OR for the effectiveness of therapies into subgroups of pharmacotherapy, non-pharmacotherapy and combination therapy. Studies that included multiple interventions or more than 1 study arm, were noted with “a number” after the same author's name (e.g., Author X, Year −1; Author X, Year −2; Author X, Year −3; etc). To avoid double counting of the reference groups, we used a “combination method” and a “splitting method.”^30,31^ Subgroup analysis was done to explore heterogeneity. Incidence was pooled into the following subgroups: study design (RCT, Cohort studies), intervention group (Pharmacological, nonpharmacological, both), length of follow-up (<1 year, 1–2 years, > 2 years), date of study (1990–2000, 2001–2005, 2006–2010, 2010–2015, ≥2016), intervention type, quality of study (≥85% score). Analysis was conducted using R package 4.2.2. (R.app GUI 1.79, S. Urbanek & H.-J. Bibiko, ^©^ R Foundation for Statistical Computing, 2021).

## Results

### Trial Flow

**Figure 1.**
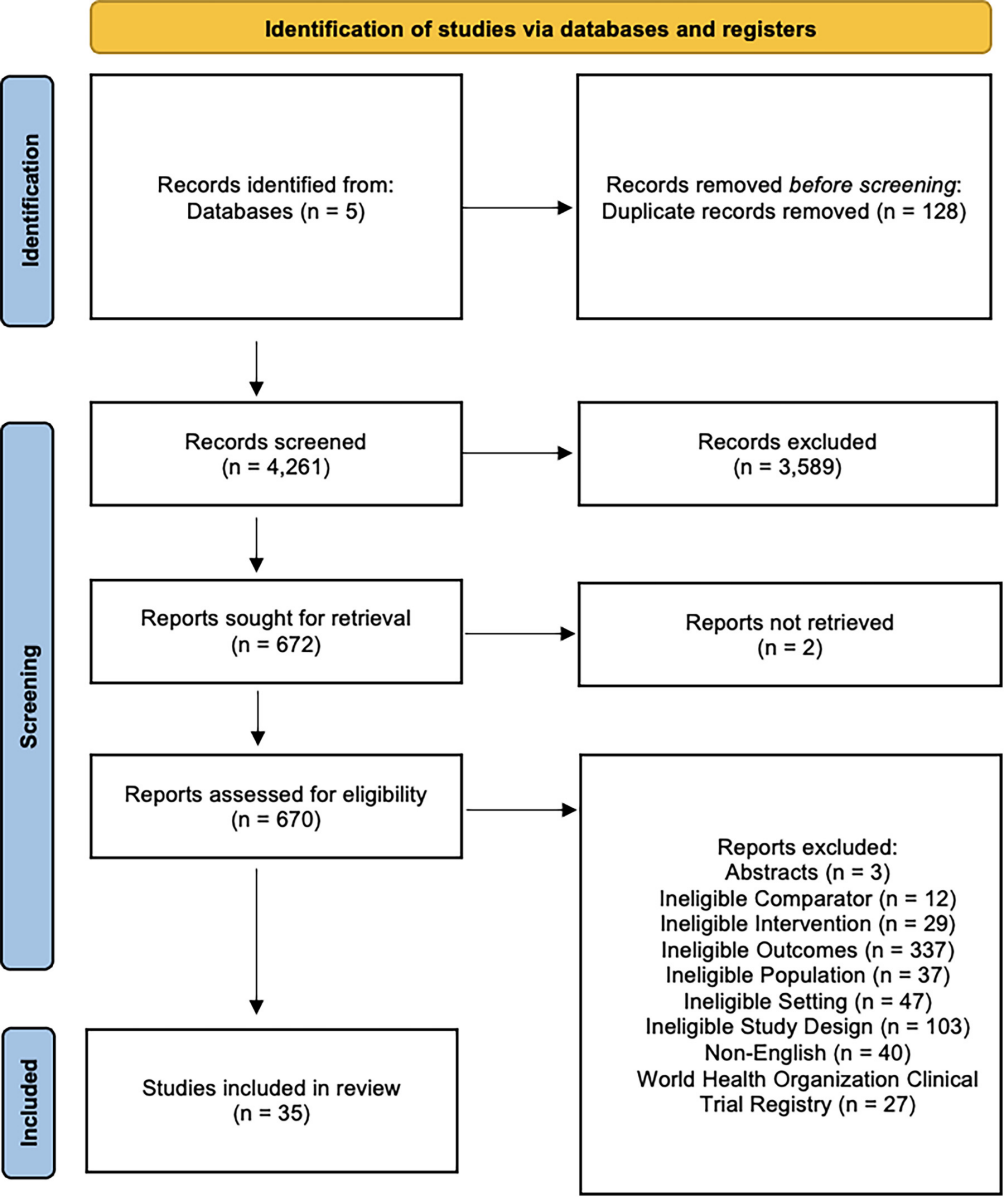
PRISMA flow diagram.

We identified 4,267 potentially relevant citations and retrieved 672 papers for full-text review (Figure 1). Of these full-text review studies, 35 studies met the eligibility criteria and were included in this systematic review.

### Study Characteristics

The 35 eligible studies were conducted in the USA (*n* = 13), the UK (*n* = 7), the Netherlands (*n* = 7), Sweden (*n* = 1), Australia (*n* = 2), Canada, Italy, Denmark, Spain, and Brazil (all *n* = 1).

Most studies included a majority of female participants, and adults of any age (mean ages 44–48 years) who identified as Caucasian. There were a variety of tools used to assess the outcomes of interest. Depending on the study, the presence and the severity of depressive symptoms were assessed through the Hamilton Depression Rating Scale (HDRS), Beck Depression Inventory (BDI), Patient Health Questionnaire (PHQ-9), Structured Clinical Interview for DSM Disorders (SCID), The Composite International Diagnostic Interview (CIDI), Montgomery-Asberg Depression Rating Scale (MADRS) or a combination of these tools. Of the studies excluded, 40 studies were in non-English language. The key characteristics of the studies are summarised in Table 1. Individual study characteristics are summarized in Table 2.

**Table 1.** Demographic Table.

Study characteristics	RCT (*n* = 30)	Cohort (*n* = 4)	Case-control (*n* = 1)
Year of study			
1990–2000	2	1	0
2001–2005	7	1	0
2006–2010	7	0	0
2010–2015	4	2	1
≥2016	10	0	0
Mean age	48.5	45.0	43.8
Female %, n of studies	71 (average of their percentages, range was from 22–100%)	75 (average of their percentages, range was from 70–80%)	70
Follow-up time			
Mean	1.5	1.2	0.5
<1 year	6 (20%)	1 (25%)	1 (100%)
1–2 years	20 (66.7%)	3 (75%)	0
>2 years	4 (13.3%)	0	0
Region, *n* (%)			
Europe	18 (60%): Netherlands 7, UK 6, Australia 2, Spain 1, Denmark 1, Italy 1	2 (50%): Sweden 1, UK 1	0
North America	11 (37%): USA 10, Canada 1	2 (50%): USA 2	1 (100%): USA
South America	1 (3%): Brazil 1	0	0
Asia	0	0	0
Africa	0	0	0
Comorbidities, *n*			
Disease Stated	6 (20%)	0	1 (100%)
Unclear (reported but no definition)	2 (6.7%)	1 (25%)	0
Not stated	22 (73.3%)	3(75%)	0
Race			
White	15 (50%): White 75–99%	0	1 (100%): White 95%
Other	Unclear	1 (25%): non-Hispanic white, minorities (black, Asian, Hispanic, other)	Unclear
Not Stated	15 (50%)	3 (75%)	0
Funding			
Governmental	17 (56.7%)	3 (60%)	1 (100%)
Private	4 (14.3%)	1 (20%)	0
Not stated	6 (20%)	0	0
Unclear	3 (10%)	0	0
Depression Score			
Hamilton Depression Rating Scale (HDRS)	5 (16.7%)	0	0
Beck Depression Inventory (BDI)	4 (13.3%)	0	0
Patient Health Questionnaire (PHQ-9)	3 (10%)	1 (20%)	1 (100%)
Structured Clinical Interview for DSM Disorders (SCID)	3 (10%)	0	0
The Composite International Diagnostic Interview (CIDI)	3 (10%)	0	0
Montgomery-Asberg Depression Rating Scale (MADRS)	1 (3.3%)	0	0
Combination	7 (23.3%)	1 (20%)	0
Others	4 (13.3%)	1 (20%)	0
Not stated	0	1 (20%)	0

**Table 2.** Individual Study Characteristics.

Randomized Controlled Trials											
Author	Country	Inclusion Criteria	Exclusion Criteria	Sex (n)	Age, mean (SD)	Follow up time (years) Lost to Follow up (%)	Primary outcome (definition)	Intervention Definition	Comparision Definition		
Bielski, 2004	USA	Age 18-65 years. MDD by DSM-IV criteria. 24-item HAM-D ≥20. Use of medically accepted contraception for women of childbearing potential.	Current Axis I disorders other than MDD, or cognitive/personality disorders interfering with participation. Substance abuse/dependence within the past 6 months. Judged to be at risk of suicide. Clinically significant and unstable medical conditions. Women who were lactating. Use of depot neuroleptics within 6 months or other neuroleptics, antidepressants, or anxiolytics within 2 weeks (5 weeks for fluoxetine) prior to the study. Prior treatment with escitalopram or venlafaxine, or failure to respond to two or more adequate antidepressant trials.	Male: 83 Female: 115 (58%)	37.4 (11.95)	Follow up: 0.21 years Lost to Follow up: 24%	Change in MADRS total score	Escitalopram	Venlafaxine-XR		
Biesheuvel-Leliefeld, 2017	Netherlands	Age 18 years or older. Full or partial remission of recurrent MDD >2 months but <5 years. At least 2 or more previous MDEs.	Severe cognitive impairment, current or past mania, hypomania, or psychosis. Current alcohol or drug abuse.	Male: 75 Female: 173 (80%)	48.7 (11.7)	Follow up: 1 years Lost to Follow up: 24%	Relapse or recurrence (MDE)	Self-help preventive CT: an 8-week manualized bibliotherapy program using a self-help book with 8 modules and minimal guidance.	TAU		
Bockting, 2008	Netherlands	At least 2 MDEs in the past 5 years, by DSM-IV criteria. Currently in remission >10 weeks <2 years. HAM-D <10.	**N**ot mentioned	Male: 46 Female: 172 (79%)	44.7 (9.5)	Follow up: 2 years Lost to Follow up: 9%	Relapse (MDE, assessed with SCID)	Preventive CT (PCT): Eight weekly sessions of PCT focused on relapse prevention. (1) PCT with intermittent antidepressant use. (2) PCT with continuous antidepressant use.	TAU		
Bockting, 2009	Netherlands	At least 2 MDEs in the previous 5 years, by DSM-IV criteria. Currently in remission for >10 weeks but <2 years. HAM-D <10.	Current mania, hypomania, history of BD, any psychotic disorder (current or previous), organic brain damage, predominant anxiety disorder. Alcohol or drug misuse. Recent ECT. Recent CT or currently engaged in psychotherapy more than twice per month.	Male: 46 Female: 126 (73%)	44.65 (9.45)	Follow up: 5.5 years Lost to Follow up: 4%	Relapse (MDE, assessed with SCID)	CT + TAU: An 8-week group-based (7–12 members) program with weekly 2-hour sessions delivered by trained cognitive therapists.	TAU		
Bockting, 2018	Netherlands	At least two previous MDEs according to DSM-IV criteria. In remission >8 weeks but <2 years. Recovery achieved with antidepressant treatment; participants had been on antidepressants for at least the past 6 months.	Current mania or hypomania, history of BD, any history of psychosis (including MDD with psychotic features), predominant anxiety disorder. Current alcohol or drug abuse. Diagnosis of organic brain damage. Receiving psychological treatment more than twice per month.	Male: 100 Female: 189 (65%)	47.3 (10.3)	Follow up: 2 years Lost to Follow up: 3.5%	Relapse (MDE, assessed with SCID and monthly IDS-SR scores)	Preventive CT (PCT): Eight weekly sessions of PCT focused on relapse prevention. (1) PCT + Antidepressants: Continued maintenance antidepressants at therapeutic doses with adherence support, (2) PCT with Tapering: Antidepressants tapered over 4 weeks with an option to restart if needed.	TAU with antidepressents		
Buszewicz, 2016	UK	Age 18 years and older. At least two MDEs within the previous 3 years. Evidence of relapse. BDI-II ≥14.	Current psychotic symptoms or impaired cognitive function. Incapacitating alcohol or drug dependence	Male: 140 Female: 418 (75%)	48.4 (12.8)	Follow up: 2 years Lost to Follow up: 13%	BDI-II score	Proactive Care Program: A 24-month intervention with 10 scheduled follow-up sessions (in-person or phone) led by trained nurses. Sessions included mood monitoring, treatment review, adherence checks, and collaborative management planning. Clinical concerns were escalated to GPs for further input	TAU		
Chilvers, 2001	UK	Age 18-70 years. RDC criteria for MDD (assessed by GP using a checklist).	Psychosis or suicidal tendencies. Postnatal depression or recent bereavement. Drug or alcohol misuse.	Male: 81 Female: 242 (75%)	37.5 (11.45)	Follow up: 1 years Lost to Follow up: 35%, 37%	Relapse (deterioration in BDI score within 6 months of remission) and remission (deterioration in BDI score after 6 months of remission).	Patients were either randomized to condition or chose their preferred option. Counseling: Six sessions provided by experienced counselors following written guidelines for depression management.	Patients were either randomized to condition or chose their preferred option. GP-Provided Care: GPs followed written guidelines for routine antidepressant drug treatment.		
Conradi, 2007	Netherlands	Age 18-70 years. Currently in treatment for MDE by their GP. History of one or more major or minor depressive episodes. Met criteria for MDE at baseline or within the past 3 months. Not suffering from a life-threatening medical condition.	Psychotic disorder, BD, or dementia. Primary alcohol or drug dependency or abuse. Pregnancy or receiving treatment for depression in a specialty mental health setting.	Male: 93 Female: 174 (65%)	42.8 (11.3)	Follow up: 3 years Lost to Follow up: 13%	Time to relapse (2 consecutive weeks of MDE symptoms before recovery) and recurrence (2 consecutive weeks of MDE symptoms after recovery).	Psycho-Educational Prevention (PEP) Program: (1) PEP Alone: Included usual care (UC). (2) Psychiatrist-Enhanced PEP: Included a 1-hour consultation with a psychiatrist advising on antidepressant treatment for the GP, followed by the PEP program (3) CBT-Enhanced PEP: Included 10–12 sessions of manualized individual cognitive behavioral therapy (CBT) led by clinical psychologists, followed by the PEP program	TAU (usual care, UC)		
de Graaf, 2011	Netherlands	Age 18-65 years. Access to the Internet at home. At least mild to moderate depressive complaints (BDI-II >16) lasting 3 months or more.	Severe psychiatric co-morbidities (e.g., psychotic disorders). Alcohol or drug dependence. Current psychological treatment for depression. Continuous antidepressant use for >3 months prior to entry.	Male: 131 Female: 172 (57%)	44.9 (11.6)	Follow up: 1 years Lost to Follow up: 11.8%	Relapse (increase of ≥9 points on the BDI-II from 3 months to either 6, 9, or 12 months of follow-up).	Computerized CBT (CCBT): (1) CCBT Alone: Online self-help program (Colour Your Life, 8 sessions + 1 booster for relapse prevention) without professional assistance. (2) CCBT + TAU: Participants were advised to consult their GP for treatment following Dutch depression guidelines, which could include 4–5 biweekly consultations or antidepressant treatment if needed	TAU		
Dolberg, 2014	Denmark	Age ≥65 years. Primary diagnosis of moderate to severe MDD by DSM-IV-TR criteria. MADRS ≥22, MMSE ≥24. Duration of MDE: At least 4 weeks. No unstabilized serious illnesses based on medical history, physical examination, electrocardiogram, and clinical laboratory tests. Patients must have given informed consent.	Current or past manic or hypomanic episode, schizophrenia or psychotic disorder, mental disorders due to a general medical condition. Personality disorder that might compromise the study. Any substance abuse disorder. At risk of suicide as determined by the investigator. Oral antipsychotics within 2 months, depot antipsychotics within 6 months, or ECT, lithium, carbamazepine, valproate, or valpromide within 1 month. Antidepressants within the last week (5 weeks for fluoxetine). Resistant to two antidepressants or non-response to citalopram/escitalopram in the current or previous episodes. Patients continuing or beginning formal psychotherapy.	Male: 65 Female: 240 (79%)	73	Follow up: 0.5 years Lost to Follow up: 23.6%	Relapse (increase in MADRS score to ≥22, or lack of efficacy as judged by the investigator).	Escitalopram: Patients in remission (MADRS score ≤12) after 12 weeks of treatment were randomized to continue escitalopram or switch to placebo	Placebo		
Duffy, 2021	UK	Age 18-74 years. Experienced at least two episodes of depression. Taking antidepressants ≥9 months but well enough to consider stopping medication.	ICD-10 criteria for current depressive illness (assessed using the CIS-R). BD, psychotic illness, dementia, or terminal illness. Contraindications to medications. Women who were pregnant, planning pregnancy, or breastfeeding.	Male: 128 Female: 350 (73%)	54 (6)	Follow up: 1.08 years Lost to Follow up: 18.4%	Time to relapse (assessed with the CIS-R)	Antidepressant Continuation (Maintenance group): Participants taking citalopram (20 mg), sertraline (100 mg), fluoxetine (20 mg), or mirtazapine (30 mg) were randomized to either continue their current medication or discontinue after a tapering period.	Antidepressant discontinuation (Discontinuation group)		
Eveleigh, 2017	Netherlands	Long-term antidepressant use (≥9 months). Appropriate long-term antidepressant use per Dutch guidelines for depressive and anxiety disorders (e.g., history of ≥3 recurrent depressive episodes and/or ≥2 relapses after discontinuation).	Current treatment in a psychiatric inpatient or outpatient clinic. History of psychosis, BD, or OCD. Current substance use disorder. Use of antidepressants for conditions such as neuropathic pain.	Male: 44 Female: 102 (70%)	56 (13.6)	Follow up: 1 years Lost to Follow up: 20.5%	Proportion of participants who successfully discontinued the antidepressant after 1 year.	Tapering Program: GPs received a patient-specific letter recommending gradual antidepressant tapering, along with information on discontinuation syndrome. Patients were invited to discuss the recommendation, with no restrictions on treatment in case of relapse or a new psychiatric disorder. A return slip was used to confirm patient intention to comply, and reasons for non-compliance were requested if applicable.	TAU		
Fava, 1998	Italy	MDD per Research Diagnostic Criteria (RDC). History of ≥3 MDEs, with the most recent episode occurring no more than 2.5 years prior to the current episode. Minimum 10-week remission between the index and preceding episode, defined as ≤2 mild symptoms with no functional impairment per RDC. Minimum global severity score of 7 for the current depressive episode.	History of manic, hypomanic, or cyclothymic features. History of active drug or alcohol abuse or dependence, or personality disorder per DSM-IV criteria. History of antecedent dysthymia.	Male: 16 Female: 24 (60%)	46.9 (11.2)	Follow up: 2 years Lost to Follow up: Not mentined	Relapse (RDC-defined MDE).	Pharmacotherapy tapering and CBT (10 biweekly 30-minute sessions). Antidepressant tapering: 25 mg amitriptyline equivalent reduced every 2 weeks until complete withdrawal (final 2 sessions were drug-free).	Pharmacotherapy and TAU		
Frank, 2007	USA	Age 20–60 years. Experiencing at least their second MDE per RDC. The immediately preceding episode occurred 10–130 weeks before the onset of the index episode. HAM-D ≥15. RSDS ≥7.	Comorbid Axis I disorders (except anxiety disorders), hypomania or mania, adult-onset dysthymia, and eating disorder not otherwise specified. Antisocial or borderline personality disorder. Substance abuse or dependence within the past 2 years. Any medical condition (excluding pregnancy) incompatible with the use of SSRIs.	Male: 0 Female: 203 (100%)	37.7 (10.1)	Follow up: 2 years Lost to Follow up: 26%	Relapse (not specified)	IPT alone: Acute Phase (12–24 weeks): Weekly IPT sessions. If not 33% reduction in HAM-D after 4 weeks, sessions increased to twice weekly for 4 weeks. Remission defined as 3 consecutive weeks with HAM-D ≤7. Maintenance Phase (2 years): Patients sustaining remission for 5 weeks randomized to weekly, twice-monthly, or monthly IPT booster sessions.	IPT with SSRI		
Frank, 1990	USA	Age 21-65 years. Experiencing at least their third MDE, with the immediately preceding episode occurring no more than 2.5 years before the onset of the present episode. Minimum 10-week remission per RDC. HAM-D ≥15. RSDS ≥7.	Diagnosis of any other Axis I disorder, except GAD or panic disorder. Antisocial or borderline personality disorder. Any medical condition incompatible with imipramine therapy.	Male: 30 Female: 98 (76%)	40.2 (10.9)	Follow up: 3 years Lost to Follow up: 17%	Relapse (new episode of MDE with HAM-D ≥15 and RSDS ≥7, confirmed by an independent psychiatrist)	Maintenance IPT (IPT-M): IPT-M + Imipramine: including medication clinic visits for monitoring and adherence support.	IPT-M + Placebo		
Gelenberg, 2004	USA	Age 18 years or older. Physically healthy male or female outpatients. Diagnosed with single-episode or recurrent major depressive disorder without psychotic features, as per DSM-IV criteria. Duration of the current depressive episode: at least 4 weeks. Hamilton Depression Rating Scale (HAM-D) score of ≥20 at baseline, indicating at least moderate severity of depression.	Not mentioned.	Male: 32 Female: 66 (67%)	41.6 (13.5)	Follow up: 0.5 years Lost to Follow up: Not mentioned	Relapse (increase in HAM-D score ≥12, HAM-D ≥14, and CGI-I ≤2)	St. John’s wort extract	Placebo		
Hollon, 2005	USA	Ate 18-70 years. Moderate to severe MDD per DSM-IV-TR. HAM-D ≥20 for 2 consecutive weeks.	History of psychosis, BD I, primary comorbid Axis I disorder. Borderline, antisocial, or schizotypal personality disorder. Clinically significant medical disorder that precluded antidepressant treatment.	Not Available	40 (12)	Follow up: 1 years Lost to Follow up: 16%	Relapse (≥3 weeks of increased symptoms)	CT + Antidepressant Medication (ADM): ADM: Patients continued with their acute treatment psychiatrist, with sessions every 2 weeks initially and monthly thereafter. Dosage adjustments and medication changes were allowed to address adverse effects or symptom recurrence.	CT + Placebo		
Howell, 2008	Australia	Age ≥18 years. Depressive disorder per DSM-IV criteria.	Diagnosis of psychotic disorders.	Male: 24 Female: 86 (78%)	39.5	Follow up: 1 years Lost to Follow up: 14.5%	Relative risk of relapse (blinded case-note review for evidence of increased depressive symptoms, medication changes, hospital admissions, new symptoms, or suicidality).	Keeping the Blues Away: a 10-step, multimodal, skills-based depression relapse prevention program, designed for general practice, using evidence-based psychosocial strategies like problem-solving.	TAU		
Jarrett, 2001	USA	Diagnosis of nonpsychotic, recurrent MDD per DSM-IV criteria. Clear interepisode recovery (≥2 separated by ≥2 months of return to normal functioning). HAM-D ≥16.	MDD with psychotic features or other primary Axis 1 disorder. Current alcohol or drug abuse. Borderline personality disorder. Imminent suicide risk identified at triage. Presence of a contraindicated medical condition or medication use.	Male: 35 Female: 92 (72%)	42.4 (1.4)	Follow up: 2 years Lost to Follow up: 32%	Relapse (MDE as determined by blind evaluator using LIFE).	Continuation-Phase CT (C-CT): Following acute-phase CT (20 individual 50-60 minute sessions over 12-14 weeks), C-CT focused on symptom reduction and relapse prevention.	Evaluation only		
Katon, 2001	USA	Age 18-80 years. Received a new antidepressant prescription (no prior prescriptions in the past 120 days) from PCP for the diagnosis of depression or anxiety. Eligible if they met high epidemiologic risk of relapse criteria or had 4 or more residual major depressive symptoms.	Recent use of lithium or antipsychotic medication. Currently seeing a psychiatrist. CAGE ≥2. Pregnancy or nursing at the time of study.	Male: 102 Female: 284 (74%)	46 (12.6)	Follow up: 1 years Lost to Follow up: 31%	Relapse (not specified)	Relapse Prevention Program: A multifaceted intervention that included patient education, 2 visits with a depression specialist, and telephone monitoring and follow-up.	TAU		
Kellner, 2006	USA	Age 18-85 years, referred for ECT. MDD per DSM-IV criteria, with or without psychosis. Appropriate for ECT determined by psychiatrist. 24-item HAM-D ≥21. For the randomized Phase 2: Achieved remission in Phase 1 (≥60% decrease in HAM-D, HAM-D ≤10 on 2 consecutive ratings while free of psychotropic medication). Modified MMSE ≥21.	Schizophrenia, BD, dementia, delirium, or other CNS diseases likely to affect cognition or response to treatment. Substance dependence within the past 12 months. Medical conditions contraindicating ECT or nortriptyline-lithium use. ECT in the 3 months prior to phase 1.	Male: 59 Female: 125 (68%)	57.2 (16.1)	Follow up: 0.5 years Lost to Follow up: 20%	Time to relapse (HAM-D ≥16 with ≥10 point increase from remission baseline, for 2 consecutive weeks).	Electroconvulsive Therapy (ECT): Standardized ECT procedures with bitemporal electrode placement and stimulus dosing at 1.5 times the threshold. Phase 1 included acute treatments thrice weekly; Phase 2 included 10 maintenance sessions over 6 months (weekly for 4 weeks, biweekly for 8 weeks, and monthly for 2 months).	Pharmcotherapy		
Kuyken, 2015	UK	Age ≥18 years. Recurrent MDD in full or partial remission per DSM-IV criteria. History of ≥3 MDEs. Currently on maintenance antidepressant.	Current MDE. History of current or past psychosis, including BD. Current substance misuse. Organic brain damage. Antisocial behavior or self-injury. Formal concurrent psychotherapy.	Male: 99 Female: 325 (77%)	49.5 (12.5)	Follow up: 2 years Lost to Follow up: 9%	Time to relapse (MDE)	MBCT: A manualized, group-based skills training program combining mindfulness-based stress reduction and CT techniques.	TAU		
Lewis, 2021	UK	Age 18–74 years. ≥2 prior MDEs or having taken antidepressants >2 years. Taking citalopram, sertraline, fluoxetine, or mirtazapine for >9 months. Recovered from most recent MDE and well enough to consider discontinuing antidepressants.	Presence of current MDE per ICD-10 criteria. Taking other antidepressants.	Male: 129 Female: 349 (73%)	54.5 (12.5)	Follow up: 1.08 years Lost to Follow up: 8%	Relapse (new MDE assessed by retrospective CIS-R).	Maintenance Antidepressant (Maintenance group): Patients maintained their current antidepressant therapy.	Tapering and discontinuing antidepressant (Discontinuation group).		
Marques, 2013	Brazil	Age 18–65 years; Female gender. MDE with no previous antidepressant use. BDI ≥11. Initiated a new antidepressant.	Schizophrenia or significant cognitive impairment. Dependence on illicit drugs.	Male: 0 Female: 68 (100%)	42.5 (13)	Follow up: 0.25 years Lost to Follow up: Not mentioned	Relapse (not specified)	Pharmacotherapy Follow-Up (Dáder Method): A structured approach to medication follow-up using pharmacists.	TAU		
Meadows, 2014	Australia	Age 18-75 years. Recurrent MDD or BD I or II per DSM-IV criteria. >2 previous MDEs.	Current symptoms or past diagnosis of a psychotic disorder. Current eating disorder or OCD. Organic mental disorder or pervasive developmental delay. Borderline or antisocial personality disorder. Current alcohol or drug dependency. Current benzodiazepine intake exceeding 20 mg diazepam equivalent.	Male: 38 Female: 165 (81%)	48.35 (12.4)	Follow up: 2 years Lost to Follow up: 5%	Relapse (not specified)	MBCT + Depression Relapse Active Monitoring (DRAM): MBCT consisted of an initial individual session followed by 8 weekly 2-hour group sessions; optional 3-monthly 5-hour booster sessions reinforced skills.	TAU + Depression Relapse Active Monitoring (DRAM): DRAM included self-management training and monthly self-monitoring.		
Moore, 2022	Canada	Age 18-65 years. MDD without psychotic features per DSM-IV-TR, currently in remission. ≥3 previous MDEs. HAM-D <10. Cognitive reactivity (CR) or mood-activated dysfunctional beliefs score ≥8. >10 weeks free of psychotropic medication, except for a stable antidepressant dosage ≥4 weeks.	BD (past or present), schizophreniform disorders, neurocognitive disorders, substance abuse/dependence (within the past 6 months). Borderline/antisocial personality disorder. Current psychotherapy or counseling more than twice per month. Current meditation practice exceeding once per week or yoga exceeding twice per week. ECT within the past 6 months.	Male: 78 Female: 149 (66%)	39.8 (13.3)	Follow up: 1 years Lost to Follow up: 34%	Relapse and time to relapse (not specified)	(1) Relaxation Group Therapy (RGT): consists of 8 weekly 2-hour sessions focused on relaxation techniques, psychoeducation, and weekly discussions. (2) MBCT: consists of 8 weekly 2-hour sessions combining mindfulness meditation practices with CT techniques.	TAU		
Navarro, 2008	Spain	Current severe MDE with psychotic symptoms, per DSM-IV. HAM-D ≥21. MMSE >25. For follow-up, remission achieved during acute treatment.	History of mania, hypomania, or non-affective psychosis. Current substance dependence. Neurological disorders including dementia. Uncontrolled medical illness. Contraindications to study treatments.	Male: 12 Female: 21 (64%)	70.5 (3.3)	Follow up: 2 years Lost to Follow up: 24%	Time to relapse (MDE by DSM-IV criteria and HAM-D ≥16).	ECT + Nortriptyline	Nortriptyline		
Segal, 2020	USA	Age ≥18 years. ≥1 MDE. PHQ-9 = 5-9.	Schizophrenia, BD, current psychosis, organic mental disorder, or pervasive developmental delay.	Male: 112 Female: 346 (75%)	48.3 (14.9)	Follow up: 1.25 years Lost to Follow up: Not mentioned	Relapse (PHQ-9 ≥15) and remission rate.	Mindful Mood Balance (MMB) + Usual care: MMB is an online, 8-session self-administered adaptation of mindfulness-based cognitive therapy, augmented with coaching over 12 weeks, starting with a 45-minute orientation call, followed by 10-minute check-ins and weekly motivational emails or calls.	Usual care (based on Kaiser Permanente's adaptation of the STAR*D guidelines for antidepressant management)		
Sullivan, 2017	USA	Adults with MDD and advanced cancer (solid tumor or blood). MADRS >19. Initially limited to veterans in hospice care, later expanded to include outpatients with advanced cancer, with goals of care primarily palliative.	Cognitive impairment (Short Portable Mental Status Questionnaire score <7) or delirium. History or current mental disorder where psychostimulants are contraindicated (e.g., psychotic disorder, active severe substance abuse, or history of stimulant abuse). Severe insomnia. Symptom score >4 on specific items of the BPRS. HADS anxiety subscale >11. Active suicidal ideation. Treatment with a non-SSRI antidepressant.	Male: 25 Female: 7 (22%)	64.5 (14)	Follow up: 0.05 years Lost to Follow up: Not mentioned	Remission rate (not specified)	Methylphenidate 10-20 mg/d + SSRI: SSRI doses were adjusted, or reduced or discontinued if moderate to severe side effects occurred.	Placebo + SSRI		
Wilson, 2003	UK	Age ≥65 years. MDD per DSM–III–R criteria. HAM-D ≥18.	MMSE ≤11. Severe and unstable physical illness. Clinically significant alcohol misuse. Significant suicidal or delusional experiences. Use of concomitant drug treatments, including psychotropic drugs, warfarin, or anticonvulsants.	Male: 33 Female: 80 (73%)	76.7 (6.8)	Follow up: 2.1 years Lost to Follow up: 72.5%	Relapse (MDE by DSM-III-R criteria and HAM-D ≥13)	Sertraline (8-week open-label treatment phase with sertraline 50-200 mg/d, followed by a 16-20 week continuation phase. Those achieving stabilization entered a 100-week double-blind, placebo-controlled maintenance phase).	Placebo		
Cohort Studies											
Author	Country	Data source	Inclusion Criteria	Exclusion Criteria	Sex	Age, mean (SD)	Covariates	Follow up time (years) Lost to Follow up (%)	Primary outcome	Exposed Definition	Unexposed Definition
Angstman, 2015	USA	Chart review (2008-2011)	MDD or dysthymia. PHQ-9 ≥10. Providers included physicians, nurse practitioners (NPs), and physician assistants (PAs) from Family Medicine, Primary Care Internal Medicine, and Community Pediatrics and Adolescent Medicine departments within a large tertiary care referral clinic.	Diagnosis of BD.	Male: 2035 Female: 4975 (71%)	40.4	Age, gender, depression severity, depression diagnosis, race	Follow up: 0.5 years Lost to Follow up: Not specified.	Continued depression care and depression outcomes: Remission = PHQ-9 score <5. Persistent symptom = PHQ-9 ≥10.	CCM: included care managers, weekly oversight by a psychiatrist, utilization of practice guidelines, and a depression registry.	TAU
Claxton, 2000	UK	MediPlus UK Primary Care Database	Age ≥18 years. Depression diagnosis within 30 days of the index date, determined by Read clinical classification codes. No diagnosis of depression or antidepressant treatment in the 6 months prior to the index date. Prescribed SSRI: fluoxetine, paroxetine, or sertraline.	Comorbid psychiatric conditions (dementia, schizophrenia, psychosis, manic depression).	Male: 2080 Female: 5413 (72%)	43.3 (16.2)	All other variables; patient charecteristics, comorbidity, index, prescription patterns	Follow up: 1.5 years Lost to Follow up: Not specified.	Relapse or recurrence was identified by any of the following: (1) Antidepressant Re-initiation: Restarting treatment after ≥6 months without prescriptions. (2) Mental Health Events: Suicide attempt, referral to psychotherapy/psychiatrist, mental health facility admission, emergency room visit for mental health, or electroconvulsive therapy. (3) Combined Events: Both re-initiation and a mental health event.	SSRI Prescription Patterns: Four mutually exclusive cohorts were defined based on antidepressant prescription records during a 6-month treatment period: (1) <120 Days Cohort: Fewer than 120 days of SSRI treatment. (2) Switching/Augmentation Cohort: At least 120 days of treatment, with a switch in SSRI or addition of a second antidepressant. (3) Upward Titration Cohort: Continued use for at least 120 days, with an increase in dosage. (4) Stable Use Cohort (Control): At least 120 days of stable-dose use of the index SSRI.	Stable-dose SSRI use
Lilja, 2016	Sweden	Dataset and data collection from patients	History of recurrent depression (≥3 past episodes) and currently in full or partial remission.	Severe or ongoing depressive episode. Presence of a neuropsychiatric disorder. Alcohol or drug addiction. Suicide risk. Ongoing psychotherapeutic treatment or another intervention study.	Male: 9 Female: 36 (80%)	49 (1.01)	Not specified	Follow up: 1.2 years Lost to Follow up: 8%	Depression relapse.	MBCT: Delivered in groups (10-12 participants) with 2.5-hour sessions over eight weeks.	TAU
Lin, 1998	USA	Data from 2 randomized controlled trials and telephone interviews for follow ups	Age 18-80 years. Diagnosis of definite or probable MDD. SCL Depression ≥0.75. Willingness to take antidepressant medication.	Alcohol abuse. Presence of psychotic symptoms. Diagnosis of dementia. Pregnancy. Terminal illness. Language barriers. Plans to disenroll from GHC within the next 12 months.	Male: 58 Female: 195 (77%)	47.15 (13.9)	Age, sex, study group (psychiatrist- or psychologist-collaboration), intervention status, and Chronic Disease Score	Follow up: 1.6 years Lost to Follow up: Not specified	Relapse (within 6 months of remssion) or recurrence (6 months or later since remission) defined as: (1) MDE at the 19-month assessment. (2) ≥2 weeks of sadness or depression with additional symptoms like sleep trouble or energy loss) between 7 and 19 months.	CCM: 3-month acute phase consisting of psychiatrist or psychologist collaboration, with continued monitoring of medication adherence in the continuation phase.	TAU
Case Control Studies											
Author	Country	Data source	Inclusion Criteria	Exclusion Criteria	Sex	Age, mean (SD)	Covariates	Follow up time (years) Lost to Follow up (%)	Primary Outcome	Case Definition	Control Definition
Angstman, 2013	USA	Patient records	MDD or dysthymia. PHQ-9 ≥10. Enrollment into Collaborative Care Management (CCM) initiated by the clinician.	Not specified.	Male: 160 Female: 368 (70%)	43.8	Age, gender, marital status (married or not), race, clinical diagnosis, baseline PHQ-9, GAD-7, AUDIT, and MDQ scores, and insurance coverage	Follow up: 0.5 years Lost to Follow up: Not specified	Prolonged care management (PCM >1 year) vs. remission at 6 months to identify patients who did not benefit from the program after one year of treatment.	CCM Program	TAU

### RCTs

Of the 35 included studies, 30 were RCTs. The majority of included studies evaluated psychological therapies: CBT;^32–39^ mindfulness-based cognitive therapy (MBCT);^40–43^ and interpersonal psychotherapy (IPT).^44,45^ The next largest group of studies evaluated pharmacologic therapies and methods; specific therapies;^46–50^ and method of therapy approach.^51–56^ There were also studies evaluating service-level programs such as prevention programs.^57–59^ Most of the comparators were “treatment as usual,” with some as “an active comparator” and a few interventions were compared to both, “active comparator” and “treatment as usual.” Follow-up times for the majority of the studies were between 1 and 2 years.

### Observational Studies

We included 4 cohort studies and 1 case-control study. Included exposures were collaborative care management,^60–62^ method of therapy approach,^63^ and mindfulness-based cognitive therapy.^64^ Most of the controls were “treatment as usual”^60–62,64^ and 1 study included “active controls.”^63^ Follow-up times for the majority of the cohort studies were between 1 and 2 years, with 6 months for the case-control study.

### Risk of Bias

Overall, the reviewed studies were of sufficient quality to be synthesized in this review. Quality appraisal scores for RCTs ranged from 73% to 96%, except for 1 study that scored 69%. The quality appraisal scores for cohort studies ranged from 91% to 95%, and for the 1 case-control study quality score was 85%. Quality appraisal results are summarised in Details of included studies are shown in Supplement File 2.

### Meta-Analysis

1. MDD relapse incidence rate:

MDD relapse incidence rate was meta-analyzed into the following subgroups:

#### Interventions

**Figures 2.**
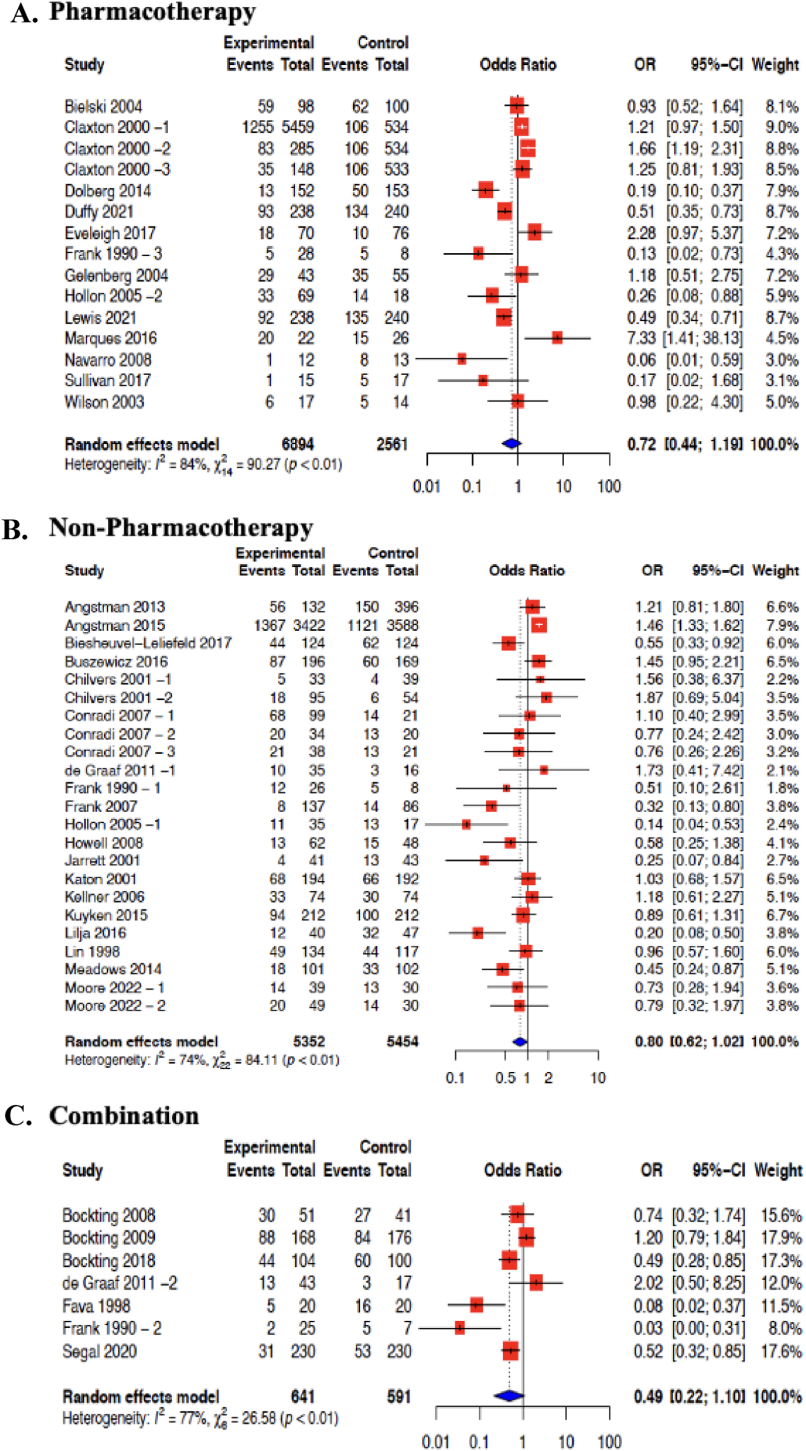
Forest plot for MDD relapse incidence rate in primary care by intervention group: (A) pharmacotherapy; (B) non-pharmacotherapy; (C) combination.

Incidence proportions were subgrouped into pharmacotherapy, non-pharmacotherapy and studies that included a combination of both group interventions. There were 13 studies that included pharmacotherapy that were meta-analyzed in this review (Figure 2 (A)). This resulted in an MDD relapse incidence rate of 37% (95% CI 28%–47%, I2 = 97%). Non-pharmacotherapies were reported in 19 studies, that were also meta-analyzed (Figure 2 (B)). These included studies showed an MDD relapse incidence rate of 36% (95% CI 30%–43%, I2 = 91%). Finally, 7 studies included a combination of both pharmacotherapy and non-pharmacotherapy interventions (Figure 2 (C)). These studies were meta-analyzed and resulted in an MDD relapse incidence rate of 39% (95% CI 27%–54%, I2 = 96%). There was “considerable heterogeneity” even after subgrouping by intervention. Pooling of MDD relapse incidence rate by specific intervention type was also done. However, this could only be done for CBT and MBCT interventions. The MDD relapse incidence rate for CBT was 42% (95% CI 32%–52%, I2 = 84%), and for MBCT was 32% (95% CI 20%–47%, I2 = 96%). There was “considerable heterogeneity” in these subgroups (Supplement File 3—Figure 4(A)).

#### Study design

Studies were also subgrouped according to study design. RCTs, including the different treatment arms within some included studies, were meta-analyzed. The MDD relapse incidence rate was 36% (95% CI 30%–42%, I2 = 93%). Cohort studies were also meta-analyzed to yield an MDD relapse incidence rate of 30% (95% CI 22%–40%, I2 = 98%). There was also “considerable heterogeneity” even after subgrouping by study design. (Supplement File 3—Figure 5(A)).

#### Length of follow-up

Incidence proportions were subgrouped according to length of follow-up of <1 year, 1–2 years, >2 years. The MDD relapse incidence rate for <1 year was 44% (95% CI 29%–62%, I2 = 96%). The MDD relapse incidence rate for 1- < 2 years was 31% (95% CI 26%–38%, I2 = 96%). And the MDD relapse incidence rate for ≥2 years was 41% (95% CI 32%–50%, I2 = 92%). There was “considerable heterogeneity” even after subgrouping by study design (Supplement File 3—Figure 6(A)).

#### Date of Study Publication

Incidence proportions were subgrouped according to the date of study publication. The MDD relapse incidence rate for studies published between 1990 and 2000, was 60% (95% CI 23%-39%, I2 = 88%). The MDD relapse incidence rate for; studies published between 2001 and 2005 was 37% (95% CI 24%-51%, I2 = 94%); studies published between 2006 and 2010 was 44% (95% CI 30%—60%, I2 = 94%); studies published between 2010 and 2015 was 35% (95% CI 23%-48%), I2 = 93%), and for studies published ≥2016 was 38% (95% CI 30%-46%, I2 = 93%). There was “considerable heterogeneity” in all subgroups (Supplement File 3—Figure 7(A)).

#### Study quality

To further explore heterogeneity, the incidence rate was pooled in studies that had a quality score of ≥85%. After pooling, the MDD relapse incidence rate was 37% (95% CI 30%–45%, I2 = 97%). There was “considerable heterogeneity” even after subgrouping by study quality (Supplement File 3—Figure 8(A)).

2.Effectiveness of therapies in MDD relapse:

MDD relapse prevention was meta-analyzed into the following subgroups:

#### Interventions

**Figures 3.**
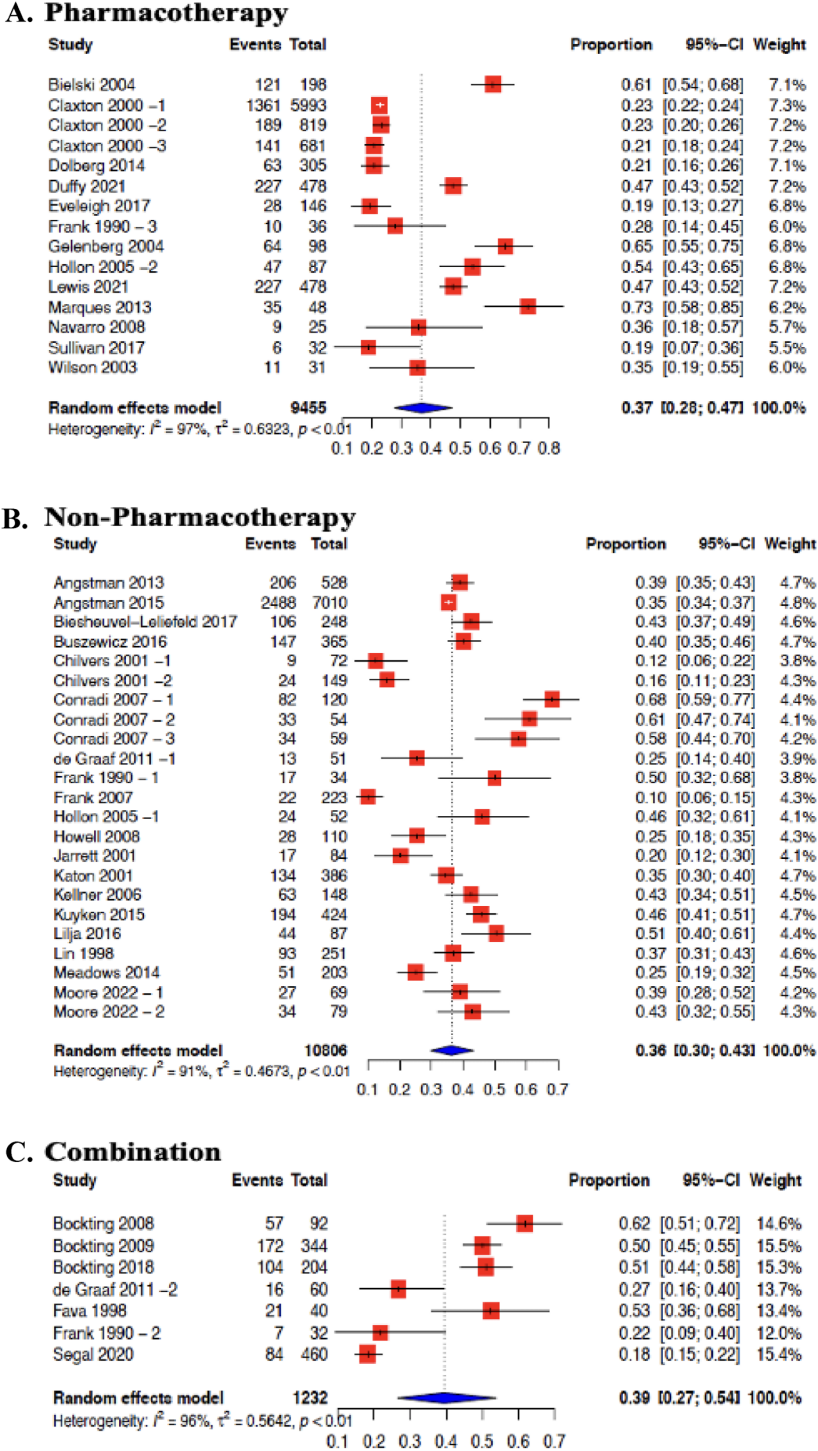
Forest plot for prevention of MDD relapse in primary care by intervention group: (A) pharmacotherapy; (B) non-pharmacotherapy; (C) combination.

Effectiveness of pharmacotherapy interventions in 13 studies were meta-analyzed (Figure 3(A)). The average risk of MDD relapse was reduced by 28% (OR = 0.72; 95% CI 0.44 to 1.19, I2 = 84%). The effectiveness of non-pharmacotherapy interventions in 19 studies was also meta-analyzed (Figure 3(B)). The average risk of MDD relapse was reduced by 20% (OR = 0.80; 95% CI 0.62–1.02, I2 = 74%). The effectiveness of the combination of both pharmacotherapy and non-pharmacotherapy interventions was also meta-analyzed from 7 studies (Figure 3(C)). The average risk of MDD relapse was reduced by 51% (OR = 0.49; 95% CI 0.22­–1.10, I2 = 77%) (Figure 3(A)). We also were able to meta-analyze studies that included a common intervention. CBT intervention effectiveness was pooled from 8 studies,^32–39^ and MBCT from 4 studies were meta-analyzed.^40–43^ All comparators were included within the same meta-analysis. Compared with all controls, the average risk of MDD relapse was reduced by 45% for CBT (OR = 0.55; 95% CI 0.30–0.99, I2 = 72%), and 35% for MBCT (OR = 0.65; 95% CI 0.45–0.94, I2 = 36%) (Supplement File 3—Figure 4 (B)).

#### Study design

Studies were subgrouped according to study design and meta-analyzed. In RCTs, the MDD relapse was reduced by 44% (OR = 0.66, 95% CI 0.52–0.84, I2 = 69%). However, when cohort studies were meta-analyzed, the MDD relapse was not different between treatment and control groups (OR = 1.05, 95% CI 0.66–1.68, I2 = 79%). There was “substantial heterogeneity” in both groups (Supplement File 3—Figure 5(B)).

#### Length of follow-up

Studies were also subgrouped according to length of follow-up of < 1 year, 1–2 years, >2 years. The MDD relapse reduction was higher with longer follows. Studies with <1-year follow-up, OR was 0.92 (95% CI 0.42–2.00, I2 = 87%); 1 to <2 years follow-up, OR was 0.80 (95% CI 0.60–1.07, I2 = 77%); and ≥2 years follow-up, OR was 0.54 (95% CI 0.36–0.80, I2 = 69%). There was “considerable heterogeneity” even after subgrouping by study design. Heterogeneity was reduced from “substantial” to “moderate” as the follow-up period increased (Supplement File 3—Figure 6(B)).

#### Date of study publication

Further subgrouping was according to the date of study publication. The OR for MDD relapse for studies published between 1990 and 2000, was 0.6 (95% CI 0.24–1.49, I2 = 78%); for studies published between 2001 and 2005, OR was 0.74 (95% CI 0.43–1.25, I2 = 58%); for studies published between 2006 and 2010, OR was 0.78 (95% CI 0.55–1.12, I2 = 44%); for studies published between 2010 and 2015, OR was 1.01 (95% CI 0.45–2.24, I2 = 89%); and for studies published ≥2016, OR was 0.63 (95% CI 0.44–0.90, I2 = 72%). Heterogeneity varied from “moderate” to “substantial” (Supplement File 3—Figure 7(B)).

#### Study quality

To explore heterogeneity even further, OR of MDD relapse was pooled in studies that had a quality score of ≥85%. After pooling, the MDD relapse OR was 0.87 (95% CI 0.66–1.16, I2 = 81%). There was “considerable heterogeneity” even after subgrouping by study quality (Supplement File 3—Figure 8 (B)).

## Discussion

This review was based on 35 studies, including 30 clinical trials, 4 cohort studies, and 1 case-control study. Overall, the studies were of high methodological quality according to the JBI Checklist. Some studies (27 studies) reported relapse of depression in primary care as the main outcome, while others (9 studies) reported it as a secondary or tertiary outcome. The included studies encompassed a variety of pharmacological and non-pharmacological interventions and exposures, differing in their source of primary care population, methods, and quality.

Our aim was to measure the MDD incidence rate in primary care and effectiveness of therapies to prevent relapse. Subgrouping according to intervention group of pharmacotherapy, non-pharmacotherapy, and combination therapy, demonstrated an MDD relapse incidence rate of these subgroups as 37%, 36%, and 39% respectively. Heterogeneity varied within each subgroup. Our review indicated a decrease in MDD relapse with therapy, as pharmacotherapy, non-pharmacotherapy, and a combination of both therapies were shown to reduce MDD relapse in primary care (28%, 20%, and 51% decrease, respectively). The meta-analysis also demonstrated that specific psychological interventions (MBCT, CBT) delivered to adults with depression may reduce the risk of experiencing relapse by 34–40%, with moderate to substantial heterogeneity.^28^ Reasons for heterogeneity might be clinical (variability in the participants, interventions), methodological (outcome measurement tools), statistical (consequence of clinical or methodological diversity, or both).^28^

To facilitate analysis and explore heterogeneity, we presented the data in detail, and pooling results into different subgroups of intervention groups, study design, length of follow-up, study publication date, and studies that had a quality score of ≥85%.

Numerous reviews have investigated depression relapse, but only a handful have specifically focused on depression within a primary care context. To the best of our understanding, a single review conducted by Gili et al.^19^ delved into depression relapse in primary care. This review recognized a small subset of studies targeting relapse prevention in primary care samples, indicating insignificant effect sizes for relapse. However, it limited its scope to RCTs reported until January 2014. In contrast, our research includes more recent studies and observational methodologies.

There have been other reviews studying depression relapse without specifically concentrating on primary care environments. Two reviews explored non-pharmacological interventions for depression relapse,^7,15^ yielding findings consistent with ours. Zhou et al.^7^ identified psychotherapy as effective in preventing relapse, while Clarke et al.^15^ suggested that psychological therapies might mitigate the risk of depression relapse after 1 year. Another review concentrated on risk factors for depression relapse,^12^ finding childhood maltreatment, residual symptoms, history of recurrence, existing or past comorbid anxiety disorders, and rumination as potential relapse predictors. Furthermore, 1 study examined risk factors contributing to increased relapse following cognitive-behavioral therapy for depression,^65^ identifying residual depressive symptoms and previous episodes as significant predictors. Another review showed that the impact of continuous antidepressant medication^66^ had a consistent effect on relapse prevention, while another reviewed the sequential combinations of pharmacotherapy and psychotherapy,^67^ linking it to reduced relapse risk. Unlike these preceding reviews, our study prioritizes depression relapse in a primary care context and does not restrict inclusion to clinical trials only.

### Strengths and Limitations of This Review

Our review was conducted in accordance with PRISMA recommendations,^20^ and possesses several methodological strengths. Firstly, we pre-registered a protocol to promote consistent conduct by the study team and ensure accountability and transparency of the completed review. Additionally, we conducted a comprehensive search of literature databases without focusing on specific interventions, ensuring all interventions within our inclusion criteria were captured. Two independent researchers scanned titles, abstracts, and full texts of studies for inclusion.^68^ We used a validated critical appraisal tool, the JBI, to assess study quality. The majority of included studies were deemed high quality by our team.

However, our study has some limitations. The generalizability of these findings might be affected for various reasons. Firstly, the reviewed studies’ samples varied in participant numbers and clinical conditions and comorbidities. Most studies excluded patients with other psychiatric diseases, personality disorders, and alcohol and drug abuse, but many did not state the possible comorbidities of the included patients. If comorbidities were mentioned, they were often not clearly defined (study characteristics table). Moreover, while participants were all from primary care setting, most studies did not specify what constituted primary care (study characteristics table). Populations in included studies might have presented with comorbidities as they were primary care patients, potentially modifying the association between depression relapse and specific interventions and exposures. Exploring effect modification was beyond the scope of this study. Furthermore, we only included English-language studies, which might affect generalizability. However, only 6% of the full-text studies were non-English, and others have reported that excluding non-English studies does not substantially influence results.^69^

Another limitation is the lack of uniformity and definition of diagnosis and tools used to identify depression relapse across studies, which may introduce bias. There is a lack of uniformity and reliability in depression measurement scales used in clinical practice, potentially causing bias.^70^ Our review included a variety of tools to assess MDD relapse and therapy effectiveness in preventing relapse. Nevertheless, all included studies clearly specified the measuring tools used to assess the outcome and how it was captured.

It is important to mention that some of the included studies were not specifically designed to quantify depression relapse (9 studies), however, relevant data from these studies were included in our review and meta-analysis. Moreover, while statistical heterogeneity was observed in the meta-analysis, it is important to acknowledge that a portion of this variability may stem from clinical heterogeneity across studies. Differences in participant characteristics, intervention protocols, and outcome measurement methods likely contributed to variations in effect sizes. This can be seen in the summary of individual study characteristics in Table 2*.* These clinical differences, while contributing to statistical heterogeneity, provide valuable insights into the potential influence of real-world variations on the observed intervention effects.^28^

### Implications for Future Research

This review highlights significant concerns that future research should consider. More data should be specified in research on depression relapse when conducting clinical or observational trials. Our review revealed that most included studies did not specify comorbidities in included patients, standard depression relapse definitions, time to relapse, and adjustment of covariates and confounders in reporting estimates (particularly in observational studies). Understanding the role of these factors could improve our knowledge of depression relapse in primary care. Moreover, our review indicated a scarcity of well-designed studies, specifically observational studies, with long follow-up times to study depression relapse in primary care. Additionally, most studies included a majority of Caucasian participants. Future clinical trials should involve racialized communities to determine if there are outcome differences based on race. These findings highlight the potential of combination therapy, encompassing pharmacological and non-pharmacological approaches, as an effective strategy to prevent relapse in MDD. While current evidence supports its efficacy, practical implementation in primary care requires careful consideration of factors such as resource availability and individual patient preferences which may influence the feasibility of implementing combination therapies in primary care. Clinicians can draw on these results to guide decisions.

## Conclusion

This review aimed to synthesize the best available evidence regarding MDD relapse incidence rate and the effectiveness of pharmacological and nonpharmacological therapies in adult patients in a primary care setting. Our findings indicate that various interventions could be used for MDD relapse in primary care, with pharmacotherapy, non-pharmacotherapy and a combination of showing preventive effects. Further research into specific pharmacological and non-pharmacological therapies, separately or in combination, with longer follow-up durations, is necessary to understand the long-term impact of these interventions on MDD relapse in primary care.

## Supplemental Material

sj-docx-1-cpa-10.1177_07067437251322401 - Supplemental material for Incidence of Major Depressive Disorder Relapse and Effectiveness of Pharmacologic and Psychological Interventions in Primary Care: A Systematic Review and Meta-Analysis: Incidence de la rechute du trouble dépressif majeur et efficacité des interventions pharmacologiques et psychologiques en soins primaires : revue systématique et méta-analyse Supplemental material, sj-docx-1-cpa-10.1177_07067437251322401 for Incidence of Major Depressive Disorder Relapse and Effectiveness of Pharmacologic and Psychological Interventions in Primary Care: A Systematic Review and Meta-Analysis: Incidence de la rechute du trouble dépressif majeur et efficacité des interventions pharmacologiques et psychologiques en soins primaires : revue systématique et méta-analyse by Waseem Abu-Ashour, Stephanie Delaney, Alison Farrell, John-Michael Gamble, John Hawboldt and Joanna E. M. Sale in The Canadian Journal of Psychiatry

sj-docx-2-cpa-10.1177_07067437251322401 - Supplemental material for Incidence of Major Depressive Disorder Relapse and Effectiveness of Pharmacologic and Psychological Interventions in Primary Care: A Systematic Review and Meta-Analysis: Incidence de la rechute du trouble dépressif majeur et efficacité des interventions pharmacologiques et psychologiques en soins primaires : revue systématique et méta-analyse Supplemental material, sj-docx-2-cpa-10.1177_07067437251322401 for Incidence of Major Depressive Disorder Relapse and Effectiveness of Pharmacologic and Psychological Interventions in Primary Care: A Systematic Review and Meta-Analysis: Incidence de la rechute du trouble dépressif majeur et efficacité des interventions pharmacologiques et psychologiques en soins primaires : revue systématique et méta-analyse by Waseem Abu-Ashour, Stephanie Delaney, Alison Farrell, John-Michael Gamble, John Hawboldt and Joanna E. M. Sale in The Canadian Journal of Psychiatry

sj-docx-3-cpa-10.1177_07067437251322401 - Supplemental material for Incidence of Major Depressive Disorder Relapse and Effectiveness of Pharmacologic and Psychological Interventions in Primary Care: A Systematic Review and Meta-Analysis: Incidence de la rechute du trouble dépressif majeur et efficacité des interventions pharmacologiques et psychologiques en soins primaires : revue systématique et méta-analyse Supplemental material, sj-docx-3-cpa-10.1177_07067437251322401 for Incidence of Major Depressive Disorder Relapse and Effectiveness of Pharmacologic and Psychological Interventions in Primary Care: A Systematic Review and Meta-Analysis: Incidence de la rechute du trouble dépressif majeur et efficacité des interventions pharmacologiques et psychologiques en soins primaires : revue systématique et méta-analyse by Waseem Abu-Ashour, Stephanie Delaney, Alison Farrell, John-Michael Gamble, John Hawboldt and Joanna E. M. Sale in The Canadian Journal of Psychiatry

sj-pptx-4-cpa-10.1177_07067437251322401 - Supplemental material for Incidence of Major Depressive Disorder Relapse and Effectiveness of Pharmacologic and Psychological Interventions in Primary Care: A Systematic Review and Meta-Analysis: Incidence de la rechute du trouble dépressif majeur et efficacité des interventions pharmacologiques et psychologiques en soins primaires : revue systématique et méta-analyse Supplemental material, sj-pptx-4-cpa-10.1177_07067437251322401 for Incidence of Major Depressive Disorder Relapse and Effectiveness of Pharmacologic and Psychological Interventions in Primary Care: A Systematic Review and Meta-Analysis: Incidence de la rechute du trouble dépressif majeur et efficacité des interventions pharmacologiques et psychologiques en soins primaires : revue systématique et méta-analyse by Waseem Abu-Ashour, Stephanie Delaney, Alison Farrell, John-Michael Gamble, John Hawboldt and Joanna E. M. Sale in The Canadian Journal of Psychiatry

## Abbreviations

AUDIT: Alcohol Use Disorders Identification Test
BD: Bipolar disorder
BDI: Beck Depression Inventory
BPRS: Brief Psychiatric Rating Scale
CBT: Cognitive behavioral therapy
CCM: Collaborative Care Management
CCS: Case-control studies
CGI-I: Clinical Global Impression-Improvement
CI: Confidence interval
CIDI: The Composite International Diagnostic Interview
CIS-R: Clinical Interview Schedule—Revised
CNS: Central nervous system
CS: Cohort studies
CT: Cognitive therapy
DSM-IV-TR: Diagnostic and Statistical Manual of Mental Disorders, 4th edition, text revision
ECT: Electroconvulsive therapy
GAD: Generalized anxiety disorder
GP: General practitioner
HADS: Hospital Anxiety and Depression Scale
HAM-D: Hamilton Depression Rating Scale
HDRS: Hamilton Depression Rating Scale
ICD-10: International classification of diseases
IPT: Interpersonal psychotherapy
JBI: Joanna Briggs Institute
LIFE: Longitudinal Interval Follow-up Examination
MADRS: Montgomery-Asberg Depression Rating Scale
MBCT: Mindfulness-based cognitive therapy
MDD: Major depressive disorder
MDE: Major depressive episode
MDQ: Mood Disorders Questionnaire
MeSH: Medical subject headings
MMSE: Mini Mental State Examination
MOOSE: Meta-analysis of Observational Studies in Epidemiology
OCD: Obsessive-compulsive disorder
OR: Odds ratio
PCP: Primary care provider
PHQ-9: Patient Health Questionnaire
PRISMA: Preferred Reporting Items for Systematic Reviews and Meta-Analyses
PROSPERO: International prospective register of systematic reviews
RCT: Randomized control trials
RDC: Research Diagnostic Criteria
RSDS: Raskin Depression Rating Scale
SCID: Structured Clinical Interview for DSM Disorders
SCL: Hopkins Symptom Check List
SSRI: Specific serotonin reuptake inhibitor
TAU: Treatment as usual
