# Supplementary material for: Incidence of Major Depressive Disorder Relapse and Effectiveness of Pharmacologic and Psychological Interventions in Primary Care: A Systematic Review and Meta-Analysis: Incidence de la rechute du trouble dépressif majeur et efficacité des interventions pharmacologiques et psychologiques en soins primaires : revue systématique et méta-analyse
Source: Can J Psychiatry. 2025 Mar 17;70(7):529–51. doi: 10.1177/07067437251322401 (PMC11915238; doi:10.1177/07067437251322401)
Supplement: sj-docx-2-cpa-10.1177_07067437251322401 - Supplemental material for Incidence of Major Depressive Disorder Relapse and Effectiveness of Pharmacologic and Psychological Interventions in Primary Care: A Systematic Review and Meta-Analysis: Incidence de la rechute du trouble dépressif majeur et efficac [file sj-docx-2-cpa-10.1177_07067437251322401.docx]

**Supplementary File 1: Search Strategy**

**Search Strategy**

Database: Ovid MEDLINE(R) and Epub Ahead of Print, In-Process, In-Data-Review & Other Non-Indexed Citations and Daily <1946 to October 14, 2021>

Run: October 14, 2021, and an updated search on September 7, 2022

| 1 | Depressive Disorder, Major/ | 33402 |
| --- | --- | --- |
| 2 | depress*.tw,kf. | 505072 |
| 3 | exp Recurrence/ | 192288 |
| 4 | Secondary Prevention/ | 21712 |
| 5 | (recurrence or recurrent or recurred or relapse or relapsing or relapsed or remission).tw,kf. | 832960 |
| 6 | 3 or 4 or 5 | 914882 |
| 7 | exp Primary Health Care/ | 174989 |
| 8 | Physicians, Family/ or Physicians/ or Physicians, Primary Care/ | 114689 |
| 9 | exp General Practice/ | 76620 |
| 10 | General Practitioners/ | 9004 |
| 11 | Nurse Practitioners/ or Primary Care Nursing/ or Community Health Nursing/ | 37925 |
| 12 | Office Visits/ | 7255 |
| 13 | Community Mental Health Services/ | 18874 |
| 14 | Ambulatory Care/ | 44985 |
| 15 | Community Health Services/ | 32518 |
| 16 | Community Medicine/ | 2048 |
| 17 | Family Nurse Practitioners/ | 61 |
| 18 | Nurse Specialists/ | 225 |
| 19 | Nurse Clinicians/ | 8379 |
| 20 | ((primary or basic) adj2 (healthcare or care)).tw,kf. | 162493 |
| 21 | ((general or family) adj (physician or physicians or doctor or doctors or practitioner or practitioners or practice)).tw,kf. | 110203 |
| 22 | family medicine.tw,kf. | 12070 |
| 23 | ((physician or doctor or physicians or doctors) adj (assistant or assistants or extender or extenders)).tw,kf. | 4976 |
| 24 | (nurse adj (specialist* or practitioner* or clinician*)).tw,kf. | 17174 |
| 25 | walk in clinic.tw,kf. | 282 |
| 26 | 26 advanced practice registered nurse.tw,kf. | 225 |
| 27 | first contact.tw,kf. | 2698 |
| 28 | 7 or 8 or 9 or 10 or 11 or 12 or 13 or 14 or 15 or 16 or 17 or 18 or 19 or 20 or 21 or 22 or 23 or 24 or 25 or 26 or 27 | 607365 |
| 29 | Depression/ | 133004 |
| 30 | Depressive Disorder/ | 74249 |
| 31 | Depressive Disorder, Treatment-Resistant/ | 1663 |
| 32 | 1 or 2 or 29 or 30 or 31 | 545869 |
| 33 | 6 and 28 and 32 | 1722 |

Database: Embase Search Strategy

Run: October 14, 2021, and an updated search on September 7, 2022

| 1 | 'depression'/de OR 'adolescent depression'/de OR 'agitated depression'/de OR 'atypical depression'/de OR 'chronic depression'/de OR 'endogenous depression'/de OR 'involutional depression'/de OR 'late life depression'/de OR 'major depression'/de OR 'masked depression'/de OR 'minor depression'/de OR 'mixed anxiety and depression'/exp OR 'mixed depression and dementia'/exp OR 'organic depression'/de OR 'reactive depression'/de OR 'recurrent brief depression'/de OR 'subsyndromal depression'/de OR 'treatment resistant depression'/de | 473840 |
| --- | --- | --- |
| 2 | depress*:ti,ab | 673636 |
| 3 | 1 OR 2 | 673636 |
| 4 | 'recurrent disease'/de OR 'recurrence risk'/de OR 'relapse'/de OR 'recrudescence'/de |  |
| 5 | recurrence:ti,ab OR recurrent:ti,ab OR recurred:ti,ab OR relapse:ti,ab OR relapsing:ti,ab OR relapsed:ti,ab OR remission:ti,ab | 5 |
| 6 | 'secondary prevention'/de | 6 |
| 7 | 4 OR 5 OR 6 | 1407343 |
| 8 | 'primary health care'/exp | 8 |
| 9 | 'physician'/de OR 'general practitioner'/exp | 9 |
| 10 | 'general practice'/exp OR 'family medicine'/exp | 10 |
| 11 | 'nurse practitioner'/de OR 'family nurse practitioner'/de OR 'nurse specialist'/de OR 'clinical nurse specialist'/de OR 'advanced practice nurse'/exp OR 'ambulatory care'/exp OR 'community mental health service'/exp OR 'community care'/de OR 'community health nursing'/de OR 'community medicine'/exp | 11 |
| 12 | ((primary OR basic) NEXT/2 (healthcare OR care)):ti,ab | 12 |
| 13 | ((general OR family) NEXT/1 (physician* OR doctor* OR practitioner* OR practice)):ti,ab | 13 |
| 14 | ((physician* OR doctor*) NEXT/1 (assistant* OR extender*)):ti,ab | 14 |
| 15 | (family NEXT/1 medicine):ti,ab | 15 |
| 16 | (nurse NEXT/1 (specialist* OR practitioner* OR clinician*)):ti,ab | 16 |
| 17 | 'walk in clinic':ti,ab | 17 |
| 18 | 'advanced practice registered nurse':ti,ab | 18 |
| 19 | 'first contact':ti,ab | 19 |
| 20 | #8 OR #9 OR #10 OR #11 OR #12 OR #13 OR #14 OR #15 OR #16 OR #17 OR #18 OR #19 | 20 |
| 21 | #3 AND #7 AND #20 | 21 |

Database: Cochrane Library Search Strategy

Run: October 15, 2021, and an updated search on September 7, 2022

| #1 | MeSH descriptor: [Depressive Disorder, Major] this term only | 5330 |
| --- | --- | --- |
| #2 | MeSH descriptor: [Depression] this term only | 13312 |
| #3 | MeSH descriptor: [Depressive Disorder, Treatment-Resistant] explode all trees | 486 |
| #4 | MeSH descriptor: [Depressive Disorder] this term only | 8161 |
| #5 | (depress*):ti,ab,kw (Word variations have been searched) | 93561 |
| #6 | {OR #1-#5} | 93561 |
| #7 | MeSH descriptor: [Recurrence] explode all trees | 12560 |
| #8 | MeSH descriptor: [Secondary Prevention] this term only | 3287 |
| #9 | (recurrence or recurrent or recurred or relapse or relapsing or relapsed or remission):ti,ab,kw | 128394 |
| #10 | {OR #7-#9} | 129482 |
| #11 | MeSH descriptor: [Primary Health Care] explode all trees | 7951 |
| #12 | MeSH descriptor: [Physicians, Family] this term only | 460 |
| #13 | MeSH descriptor: [Physicians] this term only | 936 |
| #14 | MeSH descriptor: [Physicians, Primary Care] this term only | 165 |
| #15 | MeSH descriptor: [General Practice] explode all trees | 2483 |
| #16 | MeSH descriptor: [General Practitioners] this term only | 310 |
| #17 | MeSH descriptor: [Nurse Practitioners] this term only | 307 |
| #18 | MeSH descriptor: [Primary Care Nursing] this term only | 33 |
| #19 | MeSH descriptor: [Community Health Nursing] explode all trees | 350 |
| #20 | MeSH descriptor: [Office Visits] this term only | 465 |
| #21 | MeSH descriptor: [Community Mental Health Services] this term only | 752 |
| #22 | MeSH descriptor: [Ambulatory Care] this term only | 3261 |
| #23 | MeSH descriptor: [Community Health Services] this term only | 1076 |
| #24 | MeSH descriptor: [Community Medicine] this term only | 38 |
| #25 | MeSH descriptor: [Family Nurse Practitioners] this term only | 2 |
| #26 | MeSH descriptor: [Nurse Specialists] this term only | 5 |
| #27 | MeSH descriptor: [Nurse Clinicians] this term only | 156 |
| #28 | (((primary or basic) NEXT/2 (healthcare or care))):ti,ab,kw | 23281 |
| #29 | (((general or family) NEXT (physician or physicians or doctor or doctors or practitioner or practitioners or practice))):ti,ab,kw | 13490 |
| #30 | ("family medicine"):ti,ab,kw | 728 |
| #31 | (((physician or doctor or physicians or doctors) NEXT (assistant or assistants or extender or extenders))):ti,ab,kw | 350 |
| #32 | ((nurse NEXT (specialist* or practitioner* or clinician*))):ti,ab,kw | 1976 |
| #33 | ("walk in clinic"):ti,ab,kw | 35 |
| #34 | ("advanced practice registered nurse"):ti,ab,kw | 11 |
| #35 | ("first contact"):ti,ab,kw | 247 |
| #36 | {OR #11-#35} | 42643 |
| #37 | {AND #6, #10, #36} | 775 |

Database: PsychInfo

Run: October 15, 2021, and an updated search on September 7, 2022

| **#** | **Query** | **Limiters/Expanders** | **Last Run Via** | **Results** |
| --- | --- | --- | --- | --- |
| S17 | S10 AND S13 AND S16 | Expanders - Apply equivalent subjects Search modes - Boolean/Phrase | Interface - EBSCOhost Research Databases Search Screen - Advanced Search  Database - APA PsycInfo | 892 |
| S16 | S14 OR S15 | Expanders - Apply equivalent subjects Search modes - Boolean/Phrase | Interface - EBSCOhost Research Databases Search Screen - Advanced Search  Database - APA PsycInfo | 70,442 |
| S15 | TI ( recurrence or recurrent or recurred or relapse or relapsing or relapsed or remission ) OR AB ( recurrence or recurrent or recurred or relapse or relapsing or relapsed or remission ) | Expanders - Apply equivalent subjects Search modes - Boolean/Phrase | Interface - EBSCOhost Research Databases Search Screen - Advanced Search  Database - APA PsycInfo | 69,566 |
| S14 | DE "Relapse (Disorders)" OR DE "Relapse Prevention" | Expanders - Apply equivalent subjects Search modes - Boolean/Phrase | Interface - EBSCOhost Research Databases Search Screen - Advanced Search  Database - APA PsycInfo | 9,940 |
| S13 | S11 OR S12 | Expanders - Apply equivalent subjects Search modes - Boolean/Phrase | Interface - EBSCOhost Research Databases Search Screen - Advanced Search  Database - APA PsycInfo | 326,775 |
| S12 | TI depress* OR AB depress* | Expanders - Apply equivalent subjects Search modes - Boolean/Phrase | Interface - EBSCOhost Research Databases Search Screen - Advanced Search  Database - APA PsycInfo | 321,692 |
| S11 | DE "Major Depression" OR DE "Late Life Depression" OR DE | Expanders - Apply equivalent subjects | Interface - EBSCOhost Research Databases Search Screen - Advanced | 136,625 |

Friday, October 15, 2021 12:21:24 PM

|  | "Reactive Depression" OR DE "Recurrent Depression" OR DE "Treatment Resistant Depression" | Search modes - Boolean/Phrase | Search  Database - APA PsycInfo |  |
| --- | --- | --- | --- | --- |
| S10 | S1 OR S2 OR S3 OR S4 OR S5 OR S6 OR S7 OR S8 OR S9 | Expanders - Apply equivalent subjects Search modes - Boolean/Phrase | Interface - EBSCOhost Research Databases Search Screen - Advanced Search  Database - APA PsycInfo | 105,343 |
| S9 | TI ( ( "walk in clinic" OR "advanced practice registered nurse" OR "ﬁrst contact" ) ) OR AB ( ( "walk in clinic" OR "advanced practice registered nurse" OR "ﬁrst contact" ) ) | Expanders - Apply equivalent subjects Search modes - Boolean/Phrase | Interface - EBSCOhost Research Databases Search Screen - Advanced Search  Database - APA PsycInfo | 1,148 |
| S8 | TI ( (nurse) W1 (specialist* OR practitioner* OR clinician*)  ) OR AB ( (nurse) W1 (specialist* OR practitioner* OR clinician*)  ) | Expanders - Apply equivalent subjects Search modes - Boolean/Phrase | Interface - EBSCOhost Research Databases Search Screen - Advanced Search  Database - APA PsycInfo | 3,689 |
| S7 | TI ( (physician* OR doctor*) W1 (assistant OR assistants OR extender OR extenders) ) OR AB ( (physician* OR doctor*) W1 (assistant OR assistants OR extender OR extenders) ) | Expanders - Apply equivalent subjects Search modes - Boolean/Phrase | Interface - EBSCOhost Research Databases Search Screen - Advanced Search  Database - APA PsycInfo | 794 |
| S6 | TI "family medicine" OR AB "family medicine" | Expanders - Apply equivalent subjects Search modes - Boolean/Phrase | Interface - EBSCOhost Research Databases Search Screen - Advanced Search  Database - APA PsycInfo | 1,989 |
| S5 | TI ( ( (general OR family) W1 (physician* OR doctor* OR practitioner* OR practice) ) ) OR AB ( ( (general OR family) W1 | Expanders - Apply equivalent subjects Search modes - Boolean/Phrase | Interface - EBSCOhost Research Databases Search Screen - Advanced Search  Database - APA PsycInfo | 21,939 |

|  | (physician* OR doctor* OR practitioner* OR practice) ) ) |  |  |  |
| --- | --- | --- | --- | --- |
| S4 | TI ( (primary OR basic) W2 (healthcare OR care) ) OR AB ( (primary OR basic) W2 (healthcare OR care) ) | Expanders - Apply equivalent subjects Search modes - Boolean/Phrase | Interface - EBSCOhost Research Databases Search Screen - Advanced Search  Database - APA PsycInfo | 37,801 |
| S3 | DE "Physicians" OR DE "Family Physicians" OR DE "General Practitioners" | Expanders - Apply equivalent subjects Search modes - Boolean/Phrase | Interface - EBSCOhost Research Databases Search Screen - Advanced Search  Database - APA PsycInfo | 32,600 |
| S2 | DE "Walk In Clinics" | Expanders - Apply equivalent subjects Search modes - Boolean/Phrase | Interface - EBSCOhost Research Databases Search Screen - Advanced Search  Database - APA PsycInfo | 124 |
| S1 | (((DE "Primary Health Care") OR (DE  "Community Mental Health Services" OR DE "Community Counseling")) OR (DE "Outpatient Treatment")) OR (DE "Family Medicine") | Expanders - Apply equivalent subjects Search modes - Boolean/Phrase | Interface - EBSCOhost Research Databases Search Screen - Advanced Search  Database - APA PsycInfo | 46,261 |

Database: ClinicalTrials.gov

Run: October 15, 2021, and an updated search on September 7, 2022

| (recurrence OR secondary prevention OR relapse OR recurrent OR relapsed OR relapsing OR remission OR recurring) AND "primary care" \| Depression | 97 |
| --- | --- |

**List of Grey Literature:**

Run: October 15, 2021, and an updated search on September 7, 2022

- The Centre for Addiction and Mental Health (CAMH)
- Public Health Agency of Canada (PHAC)
- The Canadian Agency for Drugs and Technologies in Health (CADTH)
- The Mood Disorders Society of Canada (MDSC)
- Association of Chairs of Psychiatry in Canada
- Canadian Institute for Health Information
- Canadian Mental Health Association
- Canadian Psychological Association
- Canadian Psychiatric Association
- Canadian Institutes of Health Research
- Institute for Neurosciences, Mental Health and Addiction
- National Network for Mental Health
- Schizophrenia Society of Canada
- The Canada Suicide Prevention Service
- Statistics Canada
- Health Canada
- Mental Health Research Canada
- Mental health commission of Canada
- American psychological association (APA)
- American Psychiatric Association (APA)
- World Health Organization (WHO)
- National Institute for Health and Care Excellence (NICE)
- National Institute of Mental Health
- National Alliance on Mental Illness
- American Foundation for Suicide Prevention
- Treatment Advocacy Center
- National Council for Behavioral Health
- National Coalition for Mental Health Recovery (NCMHR)
- Brain and Behavior Research Foundation
- Anxiety and Depression Association of America
- Depression and Bipolar Support Alliance (DBSA)
- Mental Health America (MHA)
- National Institute of Mental Health: Older Adults
- European Alliance Against Depression
- Mental Health Europe
- European Psychiatry Association
