# Supplementary material for: Incidence of Major Depressive Disorder Relapse and Effectiveness of Pharmacologic and Psychological Interventions in Primary Care: A Systematic Review and Meta-Analysis: Incidence de la rechute du trouble dépressif majeur et efficacité des interventions pharmacologiques et psychologiques en soins primaires : revue systématique et méta-analyse
Source: Can J Psychiatry. 2025 Mar 17;70(7):529–51. doi: 10.1177/07067437251322401 (PMC11915238; doi:10.1177/07067437251322401)
Supplement: sj-docx-3-cpa-10.1177_07067437251322401 - Supplemental material for Incidence of Major Depressive Disorder Relapse and Effectiveness of Pharmacologic and Psychological Interventions in Primary Care: A Systematic Review and Meta-Analysis: Incidence de la rechute du trouble dépressif majeur et efficac [file sj-docx-3-cpa-10.1177_07067437251322401.docx]

**Supplementary File 2: Quality Scores**

**Randomized Controlled Trials Quality Scores:**

| **Author/**  **Year** | **Criteria** | | | | | | | | | | | | | **Score**  **N (out of 26), %** |
| --- | --- | --- | --- | --- | --- | --- | --- | --- | --- | --- | --- | --- | --- | --- |
|  | **Q**  **1** | **Q**  **2** | **Q**  **3** | **Q**  **4** | **Q**  **5** | **Q6** | **Q7** | **Q**  **8** | **Q**  **9** | **Q**  **10** | **Q**  **11** | **Q**  **12** | **Q**  **13** |  |
| **Bielski**  **2004** | U | Y | Y | Y | Y | U | Y | Y | Y | Y | Y | Y | Y | 24, 92 |
| **Biesheuvel-Leliefeld 2017** | Y | Y | Y | N | Y | Y | Y | Y | Y | Y | Y | Y | Y | 24, 92 |
| **Bockting 2008** | U | U | N | U | U | U | U | Y | Y | Y | Y | Y | Y | 18, 69 |
| **Bockting 2009** | Y | U | U | U | U | U | Y | Y | Y | Y | Y | Y | Y | 21, 81 |
| **Bockting 2018** | Y | Y | Y | N | N | Y | Y | Y | Y | Y | Y | Y | Y | 22, 85 |
| **Buszewicz 2016** | Y | Y | Y | N | N | Y | Y | Y | Y | Y | Y | Y | Y | 22, 85 |
| **Chilvers 2001** | Y | U | Y | N | N | Y | Y | Y | Y | Y | Y | Y | Y | 22, 85 |
| **Conradi 2007** | Y | Y | Y | N | N | U | Y | Y | Y | Y | Y | Y | Y | 21, 81 |
| **de Graaf 2011** | Y | U | Y | N | N | U | Y | U | Y | Y | Y | Y | Y | 21, 81 |
| **Dolberg 2014** | U | Y | Y | Y | Y | U | Y | Y | Y | Y | Y | Y | Y | 19, 73 |
| **Duffy 2021** | Y | U | Y | Y | Y | Y | Y | Y | Y | Y | Y | Y | Y | 24, 92 |
| **Eveleigh 2017** | Y | U | Y | N | N | U | Y | Y | Y | Y | Y | Y | Y | 25, 96 |
| **Fava 1998** | U | U | Y | N | N | U | Y | Y | Y | Y | Y | Y | Y | 20, 77 |
| **Frank 2007** | U | U | Y | N | N | U | Y | Y | Y | Y | Y | Y | Y | 19, 73 |
| **Frank**  **1990** | U | U | U | Y | Y | U | Y | Y | Y | Y | Y | Y | Y | 19, 73 |
| **Gelenberg 2004** | Y | Y | Y | Y | U | U | Y | U | Y | Y | Y | Y | Y | 22, 85 |
| **Hollon 2005** | U | U | Y | Y | Y | Y | U | Y | Y | Y | Y | Y | Y | 23, 88 |
| **Howell 2008** | Y | U | Y | N | N | Y | Y | Y | Y | Y | Y | Y | Y | 23, 88 |
| **Jarrett 2001** | Y | U | Y | N | N | Y | Y | Y | Y | Y | Y | Y | Y | 21, 81 |
| **Katon 2001** | Y | U | Y | N | N | Y | Y | Y | Y | Y | Y | Y | Y | 21, 81 |
| **Kellner 2006** | Y | U | Y | N | N | Y | Y | Y | Y | Y | Y | Y | Y | 21, 81 |
| **Kuyken 2015** | Y | Y | Y | N | N | Y | Y | Y | Y | Y | Y | Y | Y | 21, 81 |
| **Lewis 2021** | Y | Y | Y | Y | Y | U | Y | Y | Y | Y | Y | Y | Y | 21, 81 |
| **Marques 2013** | U | U | Y | N | N | U | Y | Y | Y | Y | Y | Y | Y | 22, 85 |
| **Meadows 2014** | Y | U | Y | N | N | Y | Y | Y | Y | Y | Y | Y | Y | 22, 85 |
| **Moore 2022** | Y | U | Y | N | N | Y | Y | Y | Y | Y | Y | Y | Y | 25, 96 |
| **Navarro 2008** | Y | U | Y | N | N | U | Y | Y | Y | Y | Y | Y | Y | 19, 73 |
| **Segal 2020** | Y | U | Y | N | N | Y | Y | Y | Y | Y | Y | Y | Y | 21, 81 |
| **Sullivan 2017** | Y | Y | Y | Y | Y | U | Y | Y | Y | Y | Y | Y | Y | 21, 81 |
| **Wilson 2003** | Y | Y | Y | Y | Y | U | Y | Y | Y | Y | Y | Y | Y | 20, 77 |

**Cohort Studies Quality Scores:**

| **Author/Year** | **Criteria** | | | | | | | | | | | **Score N (out of 22), %** |
| --- | --- | --- | --- | --- | --- | --- | --- | --- | --- | --- | --- | --- |
|  | **Q1** | **Q2** | **Q3** | **Q4** | **Q5** | **Q6** | **Q7** | **Q8** | **Q9** | **Q10** | **Q11** |  |
| **Angstman, 2015** | Y | Y | Y | Y | Y | Y | Y | Y | Y | U | Y | 21, 95 |
| **Claxton, 2000** | Y | Y | Y | Y | Y | Y | Y | Y | U | U | Y | 20, 91 |
| **Lilja, 2016** | Y | Y | Y | U | U | Y | Y | Y | Y | Y | Y | 20, 91 |
| **Lin, 1998** | Y | Y | Y | Y | U | Y | Y | Y | Y | Y | Y | 21, 95 |

**Case-Control Studies Quality Scores:**

| **Author/Year** | **Criteria** | | | | | | | | | | **Score**  **(out of 20), %** |
| --- | --- | --- | --- | --- | --- | --- | --- | --- | --- | --- | --- |
|  | **Q1** | **Q2** | **Q3** | **Q4** | **Q5** | **Q6** | **Q7** | **Q8** | **Q9** | **Q10** |  |
| **Angstman, 2013** | Y | U | Y | Y | Y | Y | Y | Y | Y | Y | 17, 85 |
