# Supplementary material for: Incidence of Major Depressive Disorder Relapse and Effectiveness of Pharmacologic and Psychological Interventions in Primary Care: A Systematic Review and Meta-Analysis: Incidence de la rechute du trouble dépressif majeur et efficacité des interventions pharmacologiques et psychologiques en soins primaires : revue systématique et méta-analyse
Source: Can J Psychiatry. 2025 Mar 17;70(7):529–51. doi: 10.1177/07067437251322401 (PMC11915238; doi:10.1177/07067437251322401)
Supplement: sj-pptx-4-cpa-10.1177_07067437251322401 - Supplemental material for Incidence of Major Depressive Disorder Relapse and Effectiveness of Pharmacologic and Psychological Interventions in Primary Care: A Systematic Review and Meta-Analysis: Incidence de la rechute du trouble dépressif majeur et efficac [file sj-pptx-4-cpa-10.1177_07067437251322401.pptx]

## Slide 1
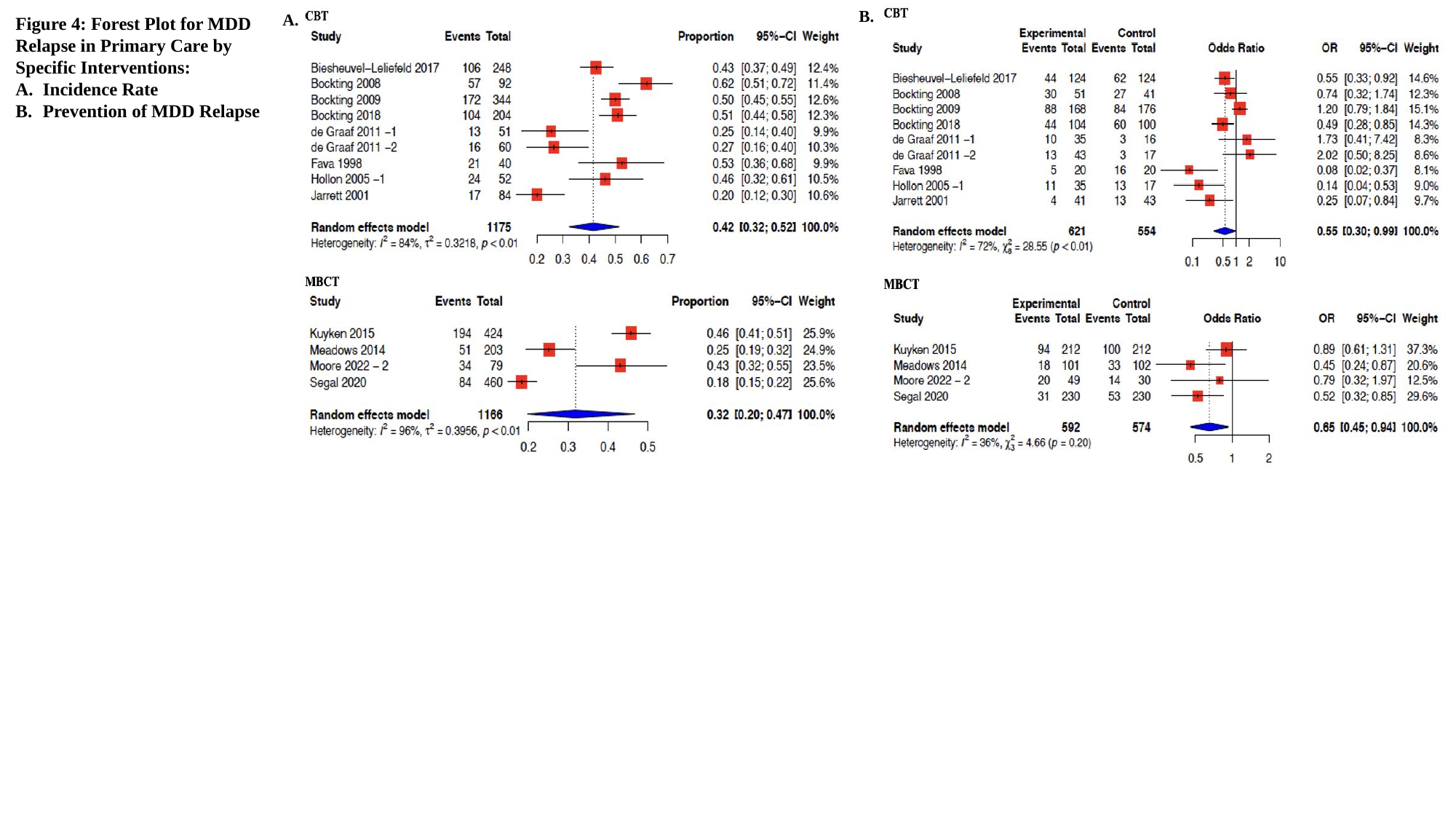

B.
A.
Figure 4: Forest Plot for MDD
Relapse in Primary Care by
Specific Interventions:
Incidence Rate
Prevention of MDD Relapse

## Slide 2
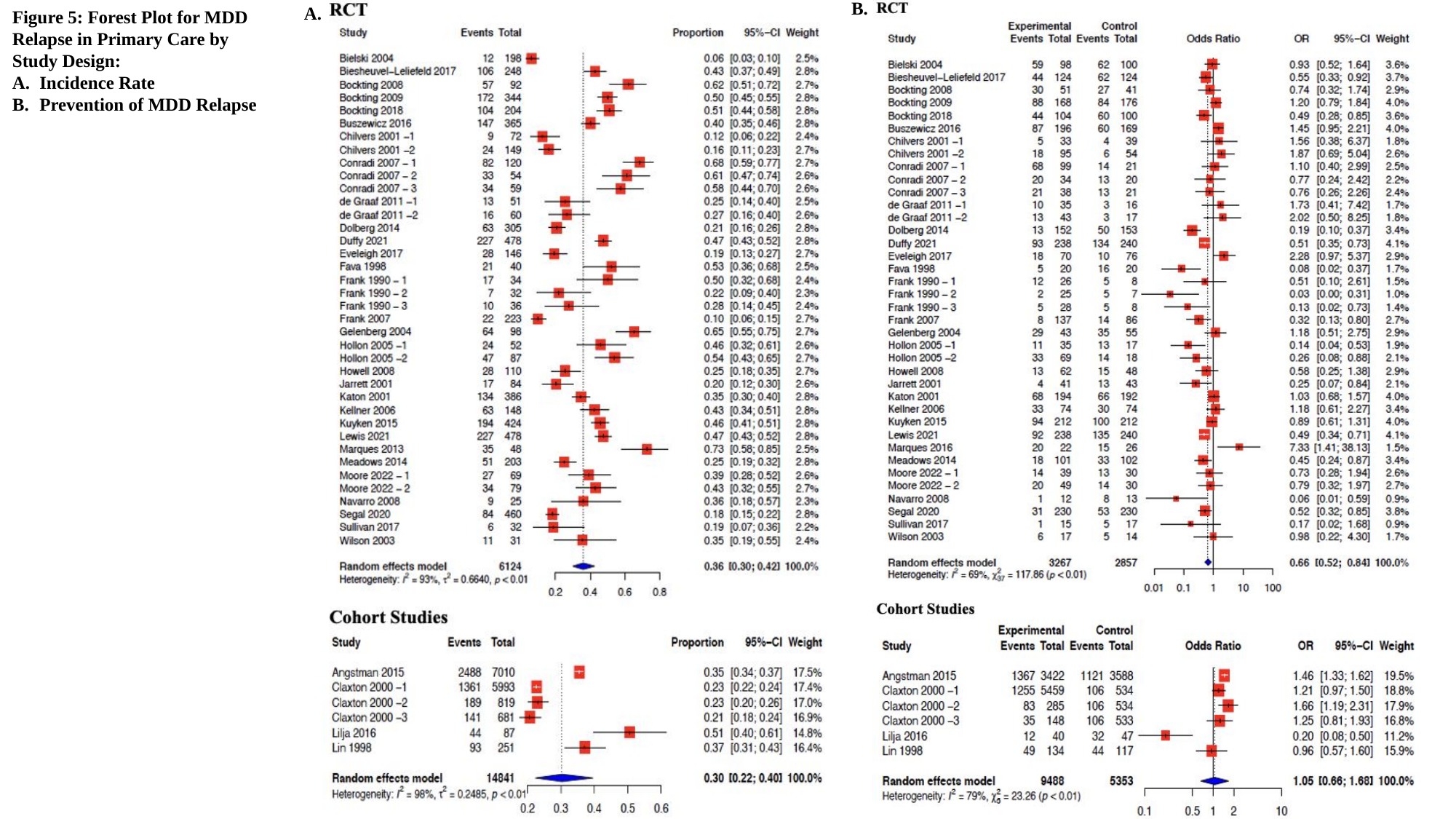

Figure 5: Forest Plot for MDD
Relapse in Primary Care by
Study Design:
Incidence Rate
Prevention of MDD Relapse
B.
A.

## Slide 3
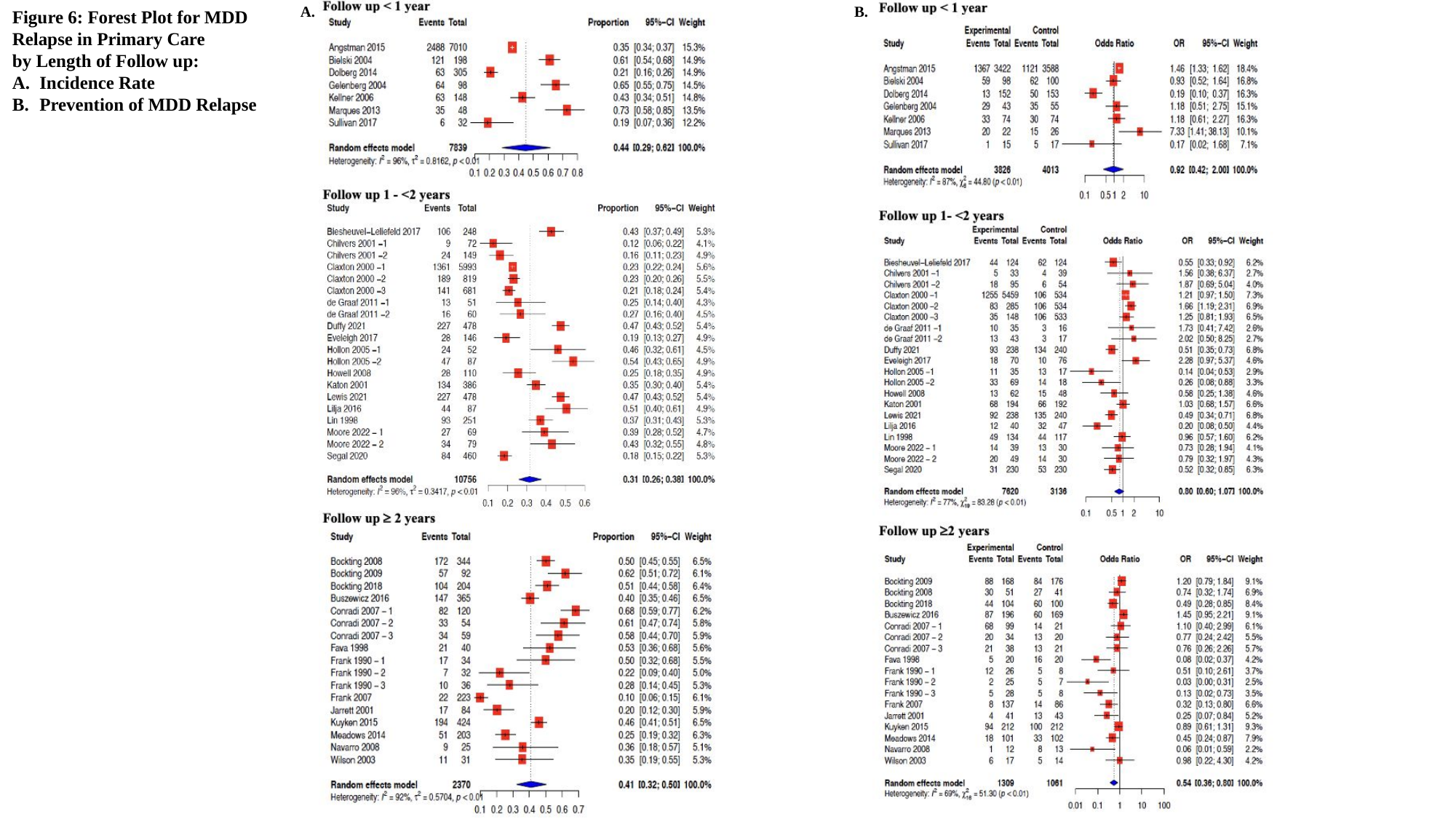

Figure 6: Forest Plot for MDD
Relapse in Primary Care
by Length of Follow up:
Incidence Rate
Prevention of MDD Relapse
A.
B.

## Slide 4
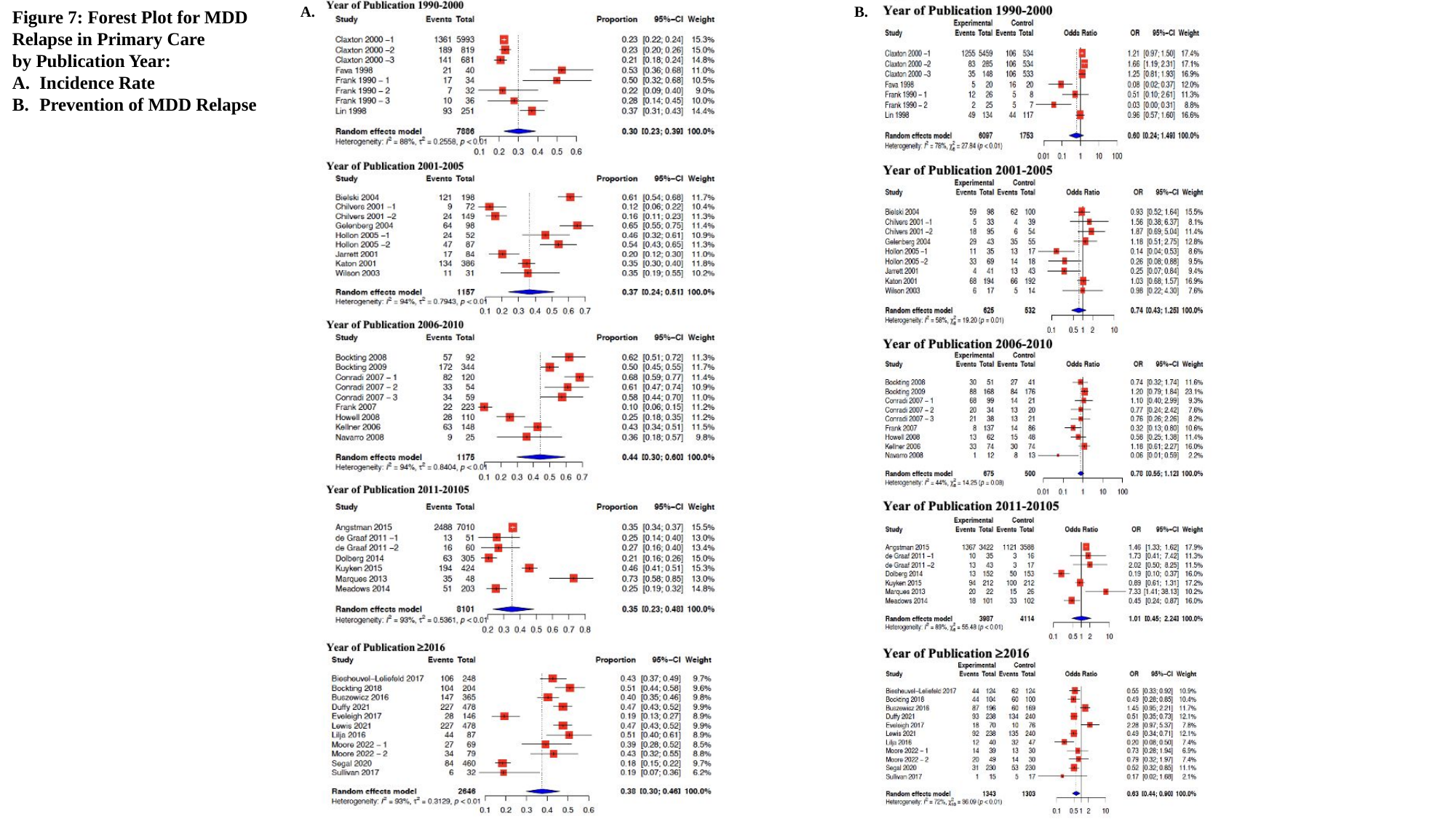

Figure 7: Forest Plot for MDD
Relapse in Primary Care
by Publication Year:
Incidence Rate
Prevention of MDD Relapse
A.
B.

## Slide 5
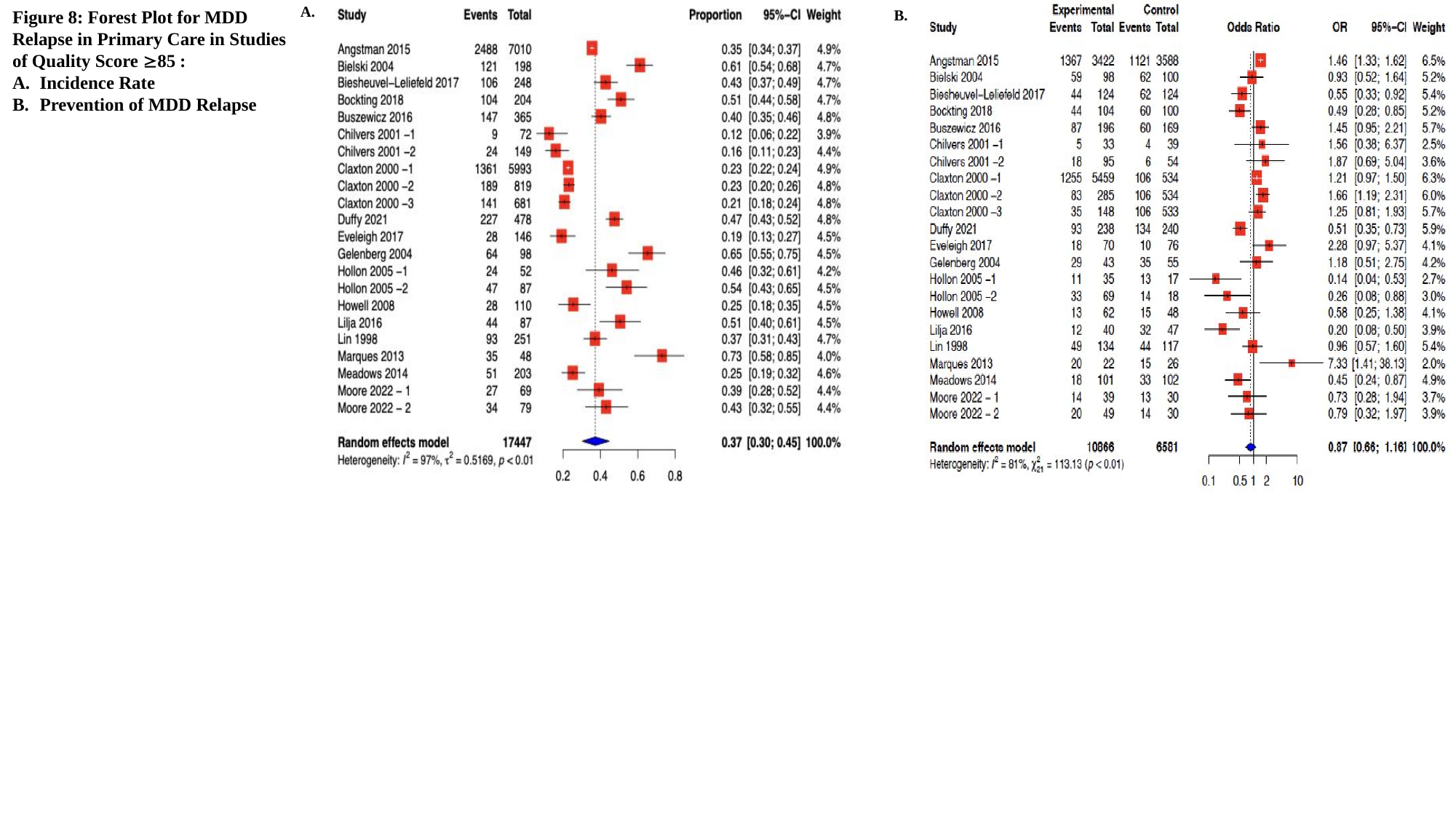

Figure 8: Forest Plot for MDD
Relapse in Primary Care in Studies
of Quality Score ≥85 :
Incidence Rate
Prevention of MDD Relapse
B.
A.
